# Ethylene-Induced Inhibition of Root Growth Requires Abscisic Acid Function in Rice (*Oryza sativa* L.) Seedlings

**DOI:** 10.1371/journal.pgen.1004701

**Published:** 2014-10-16

**Authors:** Biao Ma, Cui-Cui Yin, Si-Jie He, Xiang Lu, Wan-Ke Zhang, Tie-Gang Lu, Shou-Yi Chen, Jin-Song Zhang

**Affiliations:** 1 State Key Lab of Plant Genomics, Institute of Genetics and Developmental Biology, Chinese Academy of Sciences, Beijing, China; 2 Biotechnology Research Institute/National Key Facility for Genetic Resources and Gene Improvement, Chinese Academy of Agricultural Sciences, Beijing, China; The University of North Carolina at Chapel Hill, United States of America

## Abstract

Ethylene and abscisic acid (ABA) have a complicated interplay in many developmental processes. Their interaction in rice is largely unclear. Here, we characterized a rice ethylene-response mutant *mhz4*, which exhibited reduced ethylene-response in roots but enhanced ethylene-response in coleoptiles of etiolated seedlings. *MHZ4* was identified through map-based cloning and encoded a chloroplast-localized membrane protein homologous to *Arabidopsis thaliana* (*Arabidopsis*) ABA4, which is responsible for a branch of ABA biosynthesis. *MHZ4* mutation reduced ABA level, but promoted ethylene production. Ethylene induced *MHZ4* expression and promoted ABA accumulation in roots. *MHZ4* overexpression resulted in enhanced and reduced ethylene response in roots and coleoptiles, respectively. In root, *MHZ4*-dependent ABA pathway acts at or downstream of ethylene receptors and positively regulates root ethylene response. This ethylene-ABA interaction mode is different from that reported in *Arabidopsis*, where ethylene-mediated root inhibition is independent of ABA function. In coleoptile, *MHZ4*-dependent ABA pathway acts at or upstream of OsEIN2 to negatively regulate coleoptile ethylene response, possibly by affecting *OsEIN2* expression. At mature stage, *mhz4* mutation affects branching and adventitious root formation on stem nodes of higher positions, as well as yield-related traits. Together, our findings reveal a novel mode of interplay between ethylene and ABA in control of rice growth and development.

## Author Summary

Rice is a monocotyledonous plant that is distinct from the dicotyledonous model plant *Arabidopsis* in many aspects. In *Arabidopsis*, ethylene-induced root inhibition is independent of ABA action. In rice, however, we report here that ethylene inhibition of root growth requires ABA function. We identified *MHZ4*, a rice homolog of *Arabidopsis ABA4* that is involved in ABA biosynthesis. The *mhz4* mutant displayed reduced ABA level and exhibited ethylene-hyposensitive root, but -hypersensitive coleoptile phenotypes in etiolated seedlings. Exogenous application of ABA largely recovered the defective ethylene responses. Overexpression of *MHZ4* resulted in enhanced and reduced ethylene response in the roots and coleoptiles, respectively. In root, *MHZ4*-dependent ABA pathway genetically acts at or downstream of ethylene receptors and positively regulates root ethylene response. Moreover, ethylene treatment stimulated ABA production in roots at least through transcriptional activation of *MHZ4*. The results indicate that ethylene-induced root inhibition in rice is largely mediated through *MHZ4*-dependent ABA function. In coleoptile, *MHZ4*-dependent ABA pathway acts at or upstream of OsEIN2 and negatively regulates coleoptile ethylene response, possibly via transcriptional suppression of *OsEIN2*. Together, our findings reveal a novel mode of ethylene-ABA interaction which is fundamentally different from that in *Arabidopsis*.

## Introduction

The gaseous phytohormone ethylene regulates many aspects of plant growth and development, including seed germination, seedling growth, floral transition, sex determination, fruit ripening, organ senescence/abscission and adaptive responses to multiple biotic and abiotic stresses [1]. Ethylene is synthesized from methionine via a simple linear pathway, in which 1-aminocyclopropane-1-carboxylic acid (ACC) synthase (ACS) and ACC oxidase (ACO) function as key enzymes [2]. Ethylene is perceived by a family of membrane-bound receptors that have similarity to bacterial two-component histidine kinase and act as negative regulator of ethylene response [3]. Ethylene signal is transmitted via a linear signaling cascade that consists of CONSTITUTIVE TRIPLE RESPONSE 1 (CTR1), ETHYLENE INSENSITIVE 2 (EIN2) and EIN3/EIN3-LIKE (EIL) [4]–[6]. Ethylene receptors, CTR1 and EIN2 are all predominantly localized at the endoplasmic reticulum membranes on where they can form signaling complex [7]–[10]. Without ethylene, the receptors are believed to be active and activate CTR1 ser/thr protein kinase which in turn phosphorylates the positive regulator EIN2, likely causing the proteasomal degradation of EIN2 by F-box proteins EIN2 TARGETING PROTEIN1/2 (ETP1/2) [11]–[13]. Upon ethylene perception, EIN2 can be proteolytically cleaved and its carboxyl terminus is translocated into the nucleus, triggering EIN3/EILs-mediated transcriptional cascades to induce various ethylene responses [12], [14], [15]. EIN3/EILs are also subjected to proteasomal degradation mediated by EIN3-BINDING F-BOX PROTEIN1/2 (EBF1/2) [16], [17].

In most growth and developmental processes, ethylene achieves its function through interaction with other phytohormones [18]–[20]. Among them, ethylene extensively interacts with abscisic acid (ABA) in many biological processes [21]–[29]. ABA plays pivotal roles in seed dormancy and germination, seedling development, stomatal closure and adaptive stress responses. ABA is produced from carotenoids, and the direct precursors are xanthophylls [30]. In *Arabidopsis*, zeaxanthin is converted into violaxanthin by Zeaxanthin epoxidase ABA1 [31]. ABA4 is involved in the subsequent conversion of zeaxanthin to neoxanthin although no enzyme activity was identified [32]. In addition to ABA4, recent study revealed that *NXD1* (*Neoxanthin-Deficient 1*) is also necessary for neoxanthin synthesis but does not affect ABA accumulation in tomato and *Arabidopsis* [33]. The *cis*-isomers of both zeaxanthin and neoxanthin is then cleaved by nine-*cis*-epoxycarotenoid dioxygenase (NCED), leading to the production of xanthoxin [34]. The above steps occur in plastids. Xanthoxin is released into cytosol and converted to abscisic aldehyde by ABA2, a short-chain dehydrogenase/redutase [35]. Abscisic aldehyde oxidase (AAO) finally oxidizes the abscisic aldehyde to ABA [36]. The core components of ABA signaling pathway include ABA receptors PYR/PCAR, the negative regulator PROTEIN PHOSPHATASE 2C (PP2C) and the positive regulator SNF1- RELATED PROTEIN KINASE 2 (SnRK2). In the absence of ABA, PP2Cs such as ABSCISIC ACID INSENSITIVE1 (ABI1) repress ABA signal transduction by inhibiting SnRK2 kinase activity through removal of activating phosphates. In the presence of ABA, ABA-bound receptors inhibit PP2C activity, allowing activation of SnRK2s and subsequent phosphorylation of ABA-responsive element binding factors (ABFs) to activate ABA-responsive genes [37]–[39].

Interactions of ethylene and ABA are complicated. The two hormones interplay at multiple levels, i.e., reciprocal effects on synthesis, signaling and responsive genes [18]. Ethylene and ABA interact in both antagonistic and synergistic manners, which depend upon developmental process, organ/tissue, growth conditions and species. In root growth, ethylene and ABA synergistically inhibit root elongation. Genetic evidences revealed that ABA signaling pathway acts upstream of ethylene signaling cascade in *Arabidopsis*, as root growth of ethylene-insensitive mutants *etr1-1* and *ein2* is resistant to ABA inhibition, but the roots of ABA-insensitive mutant *abi1* and ABA-deficient mutant *aba2* display normal responses to ethylene [21], [22], [26]. Moreover, block of ABA synthesis in *ein2*, *ein3*, *ein6* or *ctr1* mutant background by introducing *aba2* mutation [26], or block of ABA signaling in the *ctr1* mutant by introducing the *abi1* mutation did not alter the ethylene response phenotypes of the respective ethylene mutants [21]. These results suggest that ABA-mediated inhibition of root growth requires functional ethylene signaling, whereas ethylene-induced root inhibition is dispensable for ABA action [21], [22]. Although the integration of ethylene and ABA signaling pathways has been elucidated in *Arabidopsis*, their interactions in other plants remain largely unclear.

Rice is an important crop worldwide. A few ethylene signalling components homologous to those of *Arabidopsis* have been characterized in rice including ethylene receptor OsETR2, OsRTH1, OsCTR and OsEIN2 [40]–[44]. Recently, through analysis of rice ethylene-response mutant *mhz7*, we find that MHZ7/OsEIN2 plays central roles in ethylene signalling and regulation of agronomic traits in rice, and clear ethylene-insensitive and hypersensitive phenotypes are identified in etiolated rice seedlings [45]. In this study, we further characterized another rice ethylene-response mutant *mhz4* (*mao huzi*, Chinese name with a English meaning of cat whiskers), which displays reduced ethylene-response in roots but enhanced ethylene-response in coleoptiles [45]. Through map-based cloning, the *MHZ4* was identified to encode a membrane protein orthologous to *Arabidopsis* ABA4, which is responsible for the conversion of zeaxanthin to neoxanthin in ABA biosynthesis pathway [32]. Mutation of *MHZ4* abolishes ABA production but promotes ethylene emission. *MHZ4* overexpression enhances root ethylene-response but reduces coleoptile ethylene-response. *MHZ4* acts downstream of ethylene receptors in regulating root growth but upstream of *OsEIN2* in regulating coleoptile elongation. Our results reveals the complicated interplay between ethylene and ABA signaling in regulating rice seedling growth and agronomic traits, providing new insight into understanding of their interaction in rice.

## Results

### Characterization of *mhz4* Mutant for Ethylene Response Phenotypes

**Figure 1:**
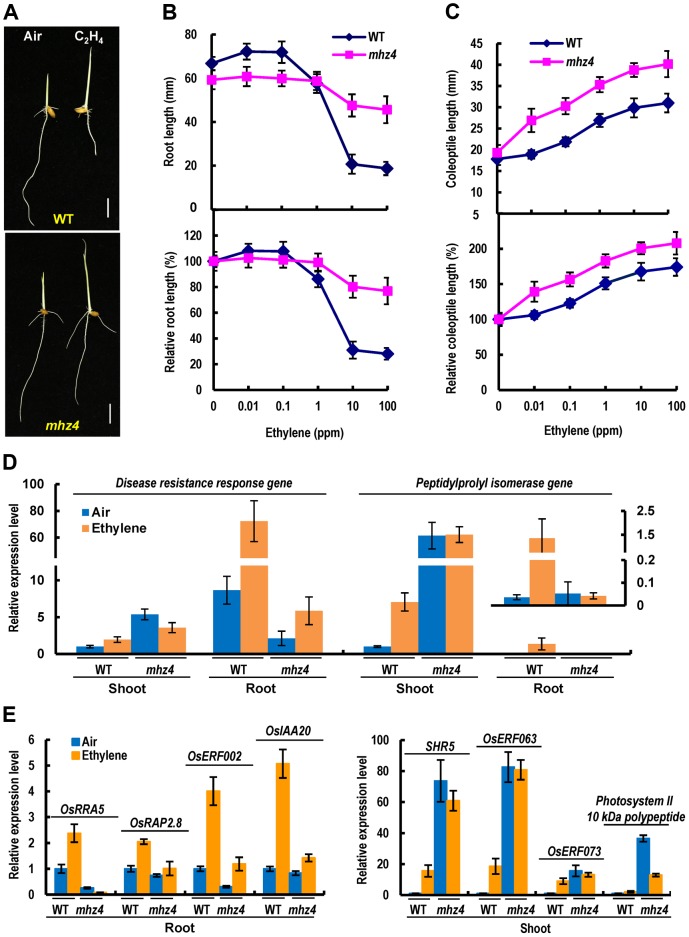
Ethylene responses in etiolated *mhz4* seedlings. Rice seedlings were grown in dark for 3 d in the presence of various concentrations of ethylene. (*A*) Ethylene-response phenotypes of WT (Nipponbare) and *mhz4* seedlings grown in air or 10 ppm ethylene. Bar = 10 mm. (*B*) Ethylene dose-response curves for root length (top) and relative root length (bottom) in WT and *mhz4* seedlings. Each point is average of 20 to 30 seedlings and bars indicate SD. (*C*) Ethylene dose-response curves for coleoptile length (top) and relative coleoptile length (bottom) of WT and *mhz4* seedlings. Others are as in (B). (*D*) Expression of ethylene-inducible genes in both shoot and root of WT and *mhz4* seedlings. Dark-grown 2 d-old seedlings were treated with or without 10 ppm ethylene for 8 h and the RNA was isolated for quantitative PCR. Data are the mean ± SD of four replicates. (*E*) Expression of ethylene-inducible genes in root (left) or shoot (right) of WT and *mhz4* seedlings. Others are as in (D).

The *mhz4* is identified previously in our screen for ethylene-response mutants in rice [45]. For dark-grown wild type (WT) seedlings, ethylene inhibited root growth but promoted coleoptile elongation in a dose-dependent manner (Figure 1A–C). The roots of etiolated *mhz4* seedlings were about 10% shorter than that of WT seedlings under normal conditions. Upon ethylene treatment, *mhz4* roots were insensitive to ethylene inhibition at lower concentrations (≤1 ppm) but displayed mild growth inhibition at higher concentrations (10 to 100 ppm), e.g. about 20% inhibition in *mhz4* compared with about 70% inhibition in the WT at 10 ppm ethylene (Figure 1A and 1B). This indicates that the roots of *mhz4* are less sensitive to ethylene. On the other hand, the coleoptiles of *mhz4* were slightly but significantly (*P*<0.004) longer than that of WT seedlings in the absence of ethylene and were much longer than that of WT under all concentrations of ethylene treatment, indicating that the coleoptiles of *mhz4* are hypersensitive to ethylene (Figure 1A and 1C). These results indicate that *mhz4* mutation oppositely affects ethylene responses in roots and coleoptiles of etiolated rice seedlings.

To further confirm the ethylene responsiveness of *mhz4* mutant, we examined expressions of ethylene-inducible genes originally identified from a chip analysis (GSE51153; [45]) and a RNA-seq analysis (SRP041468). Two genes including disease resistance response gene and peptidylprolyl isomerase gene were found to be induced by ethylene in both shoots and roots of WT seedlings (Figure 1D). Four genes (*OsERF002*, *OsRRA5*, *OsRAP2.8* and *OsIAA20*) were found to be induced mainly in roots and four genes (*SHR5*, *OsERF063*, *OsERF073* and photosystem II 10 kDa polypeptide gene) mainly in shoots of WT seedlings (Figure 1E). In *mhz4* shoots, the ethylene inducible genes were constitutively expressed at a level higher than that in ethylene-treated WT. Ethylene treatment of *mhz4* did not further increase the transcript levels probably due to their expression levels were already very high. It should be noted that we used shoots instead of coleoptiles for the gene expression analyses because similar ethylene responses were found for coleoptiles and shoots [46]. In *mhz4* roots, the transcripts of ethylene inducible genes remained at a similar or lower level when compared to those in WT in the absence of ethylene, and these genes showed no or only slight inductions compared to their ethylene inductions in WT roots (Figure 1D, E). These results indicate enhanced and reduced ethylene response in *mhz4* shoots/coleoptiles and roots, respectively, at gene expression level.

### The *MHZ4* Gene Is Homologous to *Arabidopsis ABA4*

**Figure 2:**
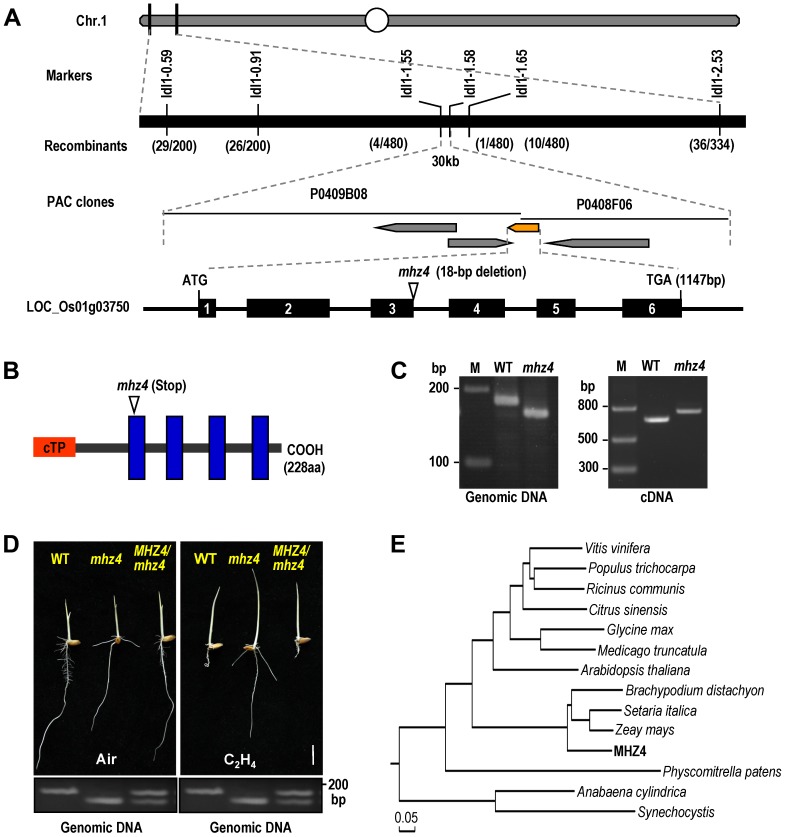
Map-based cloning of *mhz4* locus. (*A*) Fine mapping of *MHZ4* gene. The locus was mapped to chromosome 1 within a 30 Kb region between Idl1–1.55 and Idl1–1.58 markers. Mutation site is indicated in schematic diagram of *MHZ4* gene. Black boxes represent exons. (*B*) Schematic structure of the MHZ4 protein. The structure is predicted using the SMART software (http://smart.embl-heidelberg.de). Mutation site is shown. cTP indicates chloroplast transit peptide identified using the ChloroP 1.1 program (http://www.cbs.dtu.dk/services/ChloroP/). Blue columns represent transmembrane domains. (*C*) Confirmation of *mhz4* mutation site in both genomic DNA and cDNA by PCR. M: marker. (*D*) Functional complementation of *mhz4* mutant. *MHZ4* genomic DNA (4213 bp) was transformed into *mhz4* plants (*MHZ4/mhz4*), rescuing the enhanced coleoptile elongation and reduced root inhibition phenotypes of *mhz4* in the presence of ethylene (10 ppm). Bottom is confirmation of the transgene by PCR using the genomic DNA as templates. Bar = 10 mm. (*E*) Phylogenetic analysis of MHZ4/OsABA4 and its homologous proteins from other plants. The phylogenetic tree is generated by Maximum Likelihood method. Accession numbers are as follows: *Vitis vinifera*, *XP_002283875*; *Populus trichocarpa*, *XP_002305164*; *Ricinus communis*, *XP_002521222*; *Citrus sinensis*, *ADH82117*; *Glycine max*, *XP_003532342*; *Medicago truncatula*, *XP_003618901*; *Arabidopsis thaliana*, *NP_564889*; *Brachypodium distachyon*, *XP_003565288*; *Setaria italica*, *XP_004968073*; *Zeay mays*, *ACN29324*; *Physcomitrella patens*, *Pp1s108_75V6*; *Anabaena cylindrica*, *WP_015215835*; *Synechocystis*, *BAA18538*.

Genetic analysis has revealed that *mhz4* is a recessive mutation controlled by a single locus [45]. The *MHZ4* gene was identified by a map-based cloning approach using F2 plants from a cross between *mhz4* and indica rice variety Minghui 63. A total of 480 segregated mutant individuals were used for positional mapping of the *mhz4* locus. The mutation site was narrowed down to a 30-kb region in chromosome 1 between Idl1–1.55 and Idl1–1.58 markers (Figure 2A). We sequenced all four annotated genes within this region and found an 18-bp deletion in LOC_Os01g03750. The deletion occurred at the third intron and disrupted the splicing site, resulting in 81-bp un-spliced intron and causing a premature stop codon in the encoded protein (Figure 2A and 2B). The mutations were further confirmed by PCR through examination of fragment length polymorphisms in genomic DNA and cDNA of WT and *mhz4* (Figure 2C). To verify that the mutation of LOC_Os01g03750 locus is responsible for the mutant phenotype of *mhz4*, we cloned the 4213-bp DNA fragment including the complete LOC_Os01g03750 genomic sequence (1147-bp) plus 2048-bp region upstream of the start codon and 858-bp extension downstream of the stop codon from the WT, and transformed the gene into the *mhz4* plants. Ethylene response assays showed that the altered ethylene responsiveness of *mhz4* was rescued in the transgenic plants (Figure 2D). These results confirm that *MHZ4* is located at LOC_Os01g03750 locus.

The *MHZ4* gene encodes a protein of 228 amino acids that harbors an *N*-terminal signal peptide and four transmembrane domains as predicted by the SMART program (http://smart.embl-heidelberg.de/) (Figure 2B). The MHZ4 sequence shared 47% identity and 64% similarity with *Arabidopsis* ABA4 (AT1G67080), which is required for neoxanthin formation in the ABA biosynthesis pathway through an unknown mechanism [32]. Phylogenetic analysis revealed that MHZ4 protein is conserved from cyanobacteria to higher plants and is more closely related to homologues from monocotyledonous plants (Figure 2E).

### *MHZ4* Expression and Protein Subcellular Localization

**Figure 3:**
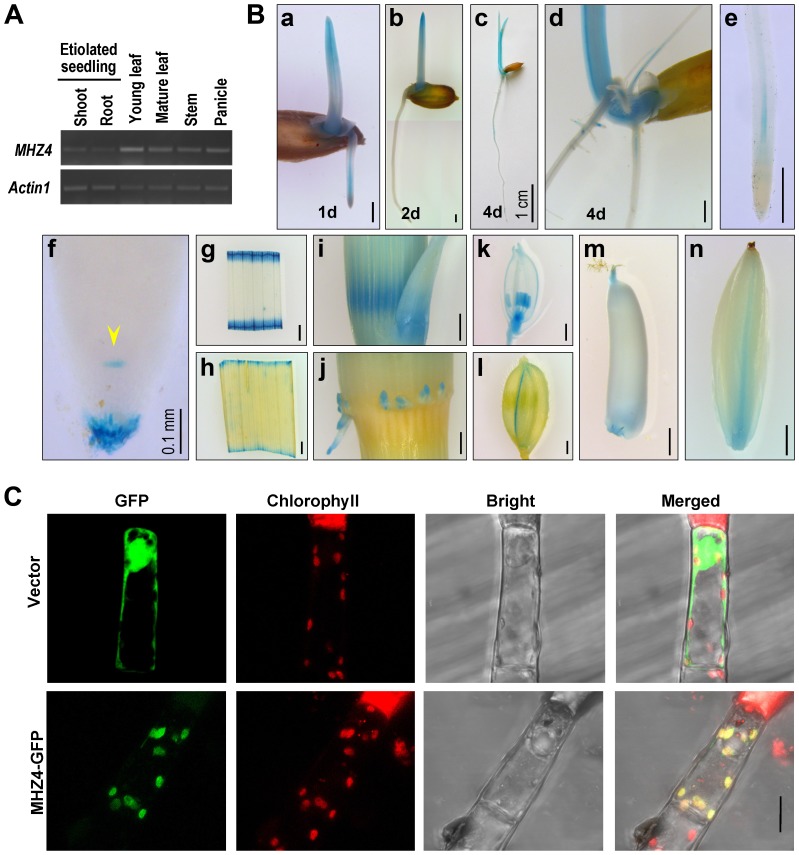
*MHZ4* expression and protein subcellular localization. (*A*) *MHZ4* expression in different rice organs detected by RT-PCR. *Actin1* was used as an internal control. (*B*) Tissue-specific expression of *MHZ4* revealed by promoter-GUS analysis. Transgenic plants expressing *MHZ4*pro::*GUS* were used for analysis. Rice organs were stained for GUS for two days. At least 10 samples for each organ were observed and representative ones are presented. (a–c) 1 d- to 4 d-old etiolated seedlings. (d) GUS signals in adventitious roots and lateral roots of 4 d-old seedlings. (e) GUS staining in vascular tissues of root tips. (f) GUS staining in quiescent center (arrow head) and root caps of root tips. (g, h) GUS staining in segments of young (g) and mature (h) leaf blades. (i) GUS staining in young stem nodes and the base of axillary buds. (j) GUS staining in adventitious roots derived from nodes. (k) GUS staining in the anthers and pistils of young flowers. (l) GUS staining in the lemma of flowers. (m) Staining in the top and bottom of an ovary. (n) GUS staining in a developing grain. Bars are 1 mm except for those indicated. (*C*) Subcellular localization of MHZ4 in chloroplasts of tobacco glandular hairs as revealed by GFP-fusion protein. The constructs were transiently expressed in tobacco leaf cells by microprojectile bombardment. GFP fluorescence was detected using confocal microscopy. Red fluorescence indicates chlorophyll. Yellow color indicates co-localization of MHZ4 with chloroplasts. Bar = 10 µm.

*MHZ4* accumulation was examined by semiquantitative RT-PCR. The transcripts were detected in all organs from vegetative to reproductive stages and found to be more abundant in young leaves (Figure 3A). Transgenic rice plants harboring *MHZ4* promoter::β-glucuronidase (*GUS*) construct were also generated and GUS staining assay was performed to evaluate the promoter activity. In etiolated seedlings, *MHZ4* expression was detected in both roots and coleoptiles, and the signals were also present in the vascular tissues of roots (Figure 3B, a to e). In root apexes, GUS signals were observed in the putative quiescent center (QC) and root caps (Figure 3B, f). In field-grown plants, *MHZ4* was expressed in leaf blades, young stem nodes, the base of axillary buds and adventitious roots derived from the nodes (Figure 3B, g to j). In reproductive organs, the expression of *MHZ4* was detected in the anthers and pistil of young flowers, lemma of mature flowers, and parts of developing grains (Figure 3B, k to n).

The MHZ4 protein was predicted to localize to the chloroplasts by using the ChloroP 1.1 program (http://www.cbs.dtu.dk/services/ChloroP/). To experimentally verify the localization, MHZ4 coding sequence was fused in frame with GFP and transiently expressed in tobacco leaves. The fluorescence signals of MHZ4-GFP fusion protein were found in the chloroplasts of tobacco glandular hairs, as identified by co-localization with the chlorophyll autofluorescence (Figure 3C). This result is consistent with the localization of Arabidopsis ABA4 in chloroplast envelope [47].

### Ethylene-Induced Root Inhibition in Rice Is Largely Mediated through *MHZ4*-Dependent ABA Accumulation

**Figure 4:**
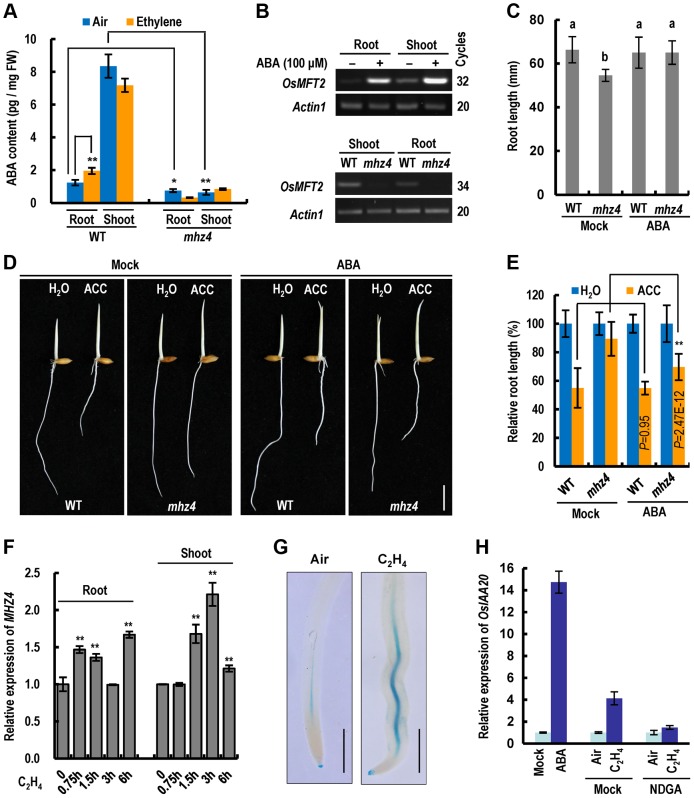
Ethylene-induced root inhibition is largely mediated through *MHZ4*-dependent ABA accumulation. (*A*) ABA levels in WT and *mhz4* seedlings in the absence or presence of ethylene. Two-day-old etiolated seedlings were treated with or without 100 ppm of ethylene for 48 h. Data are the mean ± SD of three replicates. * and ** indicate significant difference between the compared two samples at P<0.05 and P<0.01, respectively. (*B*) Expression of ABA-responsive gene *OsMFT2* (LOC_Os01g02120). Upper, *OsMFT2* transcripts in 3 d-old etiolated seedlings of WT in response to ABA (100 µM, 6 h). Bottom, *OsMFT2* transcripts detected in *mhz4* and WT etiolated seedlings. *Actin1* was used as an internal control. (*C*) Rescue of the root length of *mhz4* by ABA. Rice seedlings were grown in the dark for 2.5 days in the presence or absence (Mock) of 0.04 µM ABA. Each column is average of 40 seedlings and bars indicate SD. Different letters above each column indicate significant difference between the compared pairs (P<0.01). (*D*) Rescue of the reduced ethylene sensitivity of *mhz4* roots by ABA. WT and *mhz4* seedlings were grown in the dark for 2.5 d in the absence or presence of 10 µM ACC, with or without supplementation of 0.1 µM ABA. Bar = 10 mm. (*E*) Quantification of root inhibition in (D). Each column is average of 40 seedlings and bars indicate SD. ** indicates significant difference between the linked two samples at P<0.01. (*F*) Quantitative PCR analysis of *MHZ4* expression in response to ethylene. WT seedlings were grown in the dark for 2.5 d and then treated with 10 ppm ethylene for 0–6 h. Data are the mean ± SD of four replicates. ** indicate significant difference compared to 0 h at P<0.01. (*G*) Ethylene-induced GUS activity in roots of transgenic plants harboring *MHZ4*pro::*GUS* construct. One-day-old etiolated seedlings were treated with or without 10 ppm ethylene for 24 h. The roots were cut off and stained for GUS activity for two days. Twenty to thirty roots were observed for each treatment and representative samples are presented. Bar = 1 mm. (*H*) Quantitative PCR analysis of *OsIAA20* expression in response to ABA or ethylene+NDGA. Dark-grown 2 d-old WT seedlings were treated with 100 µM ABA for 6 h, or treated with 10 ppm ethylene for 8 h in the presence or absence (Mock) of 100 µM NDGA. The RNA from roots was isolated for quantitative PCR. Data are the mean ± SD of four replicates.

Since MHZ4 is homologous to *Arabidopsis* ABA4 in ABA biosynthesis, we tested whether endogenous ABA contents in *mhz4* were altered. The *mhz4* mutant contains 61% and 7.8% of WT ABA levels in roots and shoots, respectively, demonstrating that ABA production is severely hampered in *mhz4* mutant (Fig. 4A). Ethylene induced ABA accumulation in WT roots but not in shoots, suggesting organ-specific regulation of ABA accumulation (Fig. 4A). Ethylene induction of ABA was not observed in *mhz4* roots (Fig. 4A), indicating that MHZ4 is required for ethylene-induced ABA accumulation. We further examined the expression of ABA-responsive gene *OsMFT2* (LOC_Os01g02120) previously identified by Lenka et al. [48]. *OsMFT2* expression was dramatically induced by ABA in both roots and shoots of WT seedlings (Figure 4B, top). However, the transcripts were barely detectable in *mhz4* mutant compared with those in WT (Figure 4A, bottom). These results indicate that MHZ4 is responsible for ABA biosynthesis in rice.

Under normal growth condition, the *mhz4* roots of etiolated seedlings are slightly but significantly shorter than the WT roots (Figure 4C). Treatment of the seedlings with 0.04 µM ABA completely restored the short root phenotype (Figure 4C), suggesting that basal levels of endogenous ABA are required for the maintenance of normal root elongation. The same ABA concentration showed no obvious stimulation on root growth of WT seedlings (Figure 4C).

Considering that *MHZ4* mutation leads to the reduced ethylene sensitivity in *mhz4* roots, we investigated whether addition of ABA could rescue the ethylene response of the mutant. The 0.1 µM ABA was used in the complementation assay because at this concentration, no obvious inhibitory effects were observed on root growth in WT seedlings (Figure S1). In the presence of 10 µM ACC (precursor of ethylene), application of 0.1 µM ABA largely rescued the defective response of *mhz4* roots to ethylene (Figure 4D and 4E), indicating that reduced ethylene sensitivity of *mhz4* roots is most likely caused by the lack of ABA.

We further examined *MHZ4* expression in response to ethylene. *MHZ4* transcripts were significantly induced by ethylene in WT roots and shoots (Figure 4F). Promoter-GUS analysis also showed that ethylene treatment stimulated *MHZ4* promoter activity mainly in the vascular tissues of roots (Figure 4G). These results suggest a role for MHZ4 in root growth control. It should be noted that although *MHZ4* expression was also induced by ethylene in shoots, the ABA level did not increase after ethylene treatment (Figure 4A and 4F). This is likely owning to ethylene-activated ABA catabolism for a homeostasis in shoots [23], [30], [49].

To further elucidate the role of ethylene-triggered ABA in root ethylene response, we investigated the expression of ethylene-inducible genes in response to ABA as well as ABA biosynthesis inhibitor. *OsIAA20* transcripts were dramatically induced not only by ethylene but also by ABA in rice roots (Figure 4H). However, ethylene induction of *OsIAA20* expression was abolished in the presence of NDGA (nordihydroguaiaretic acid), an ABA biosynthesis inhibitor that specifically inhibits NCED enzyme activity (Figure 4H). These results suggest that ethylene-induced ABA mediates expression of some ethylene-responsive genes. Taken together, these findings suggest that ethylene-induced root growth inhibition is largely mediated through *MHZ4*-dependent ABA accumulation.

### Ethylene-Hypersensitivity of *mhz4* Coleoptiles Is Caused by Enhanced Ethylene Signaling

**Figure 5:**
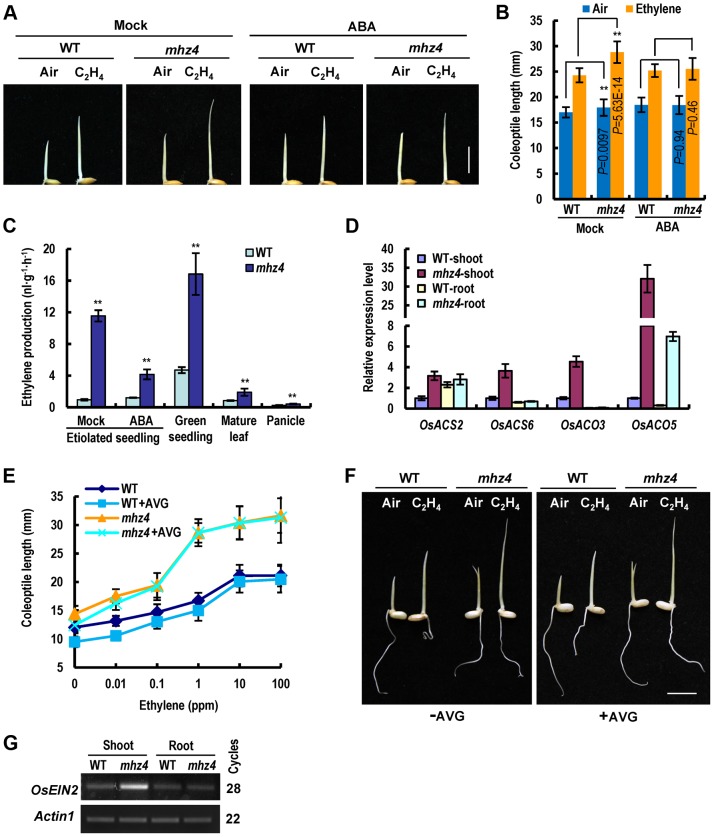
Enhanced ethylene-response in *mhz4* coleoptiles is rescued by ABA and *MHZ4* mutation leads to ethylene overproduction. (*A*) ABA rescue of enhanced ethylene-response phenotype in *mhz4* coleoptiles. WT and *mhz4* seedlings were grown in the dark for 2.5 d in the absence or presence of 10 ppm ethylene, with or without supplementation of 0.1 µM ABA. Bar = 10 mm. (*B*) Quantification of coleoptile growth with treatments in (A). Each column is average of 30 seedlings and bars indicate SD. ** indicates significant difference between the linked two samples at P<0.01. (*C*) Ethylene production in WT and *mhz4* mutant. Data are the mean ± SD of three replicates. ** indicate significant difference compared to WT at P<0.01. (*D*) Expression of ethylene biosynthetic genes in 3 d-old etiolated seedlings of WT and *mhz4*. (*E*) Ethylene dose-response curves for coleoptile elongation in WT and *mhz4* seedlings in the presence or absence of 5 µM of ethylene biosynthesis inhibitor AVG. Dark grown seedlings were treated with various concentrations of ethylene for 2.5 d. Each point is average of 25 to 30 seedlings and bars indicate SD. (*F*) Coleoptile elongation phenotypes of WT and *mhz4* in the presence or absence of 5 µM of AVG. The ethylene concentration was 10 ppm. Others are as in (E). Bar = 10 mm. (*G*) *OsEIN2* gene expression in WT and *mhz4* etiolated seedlings detected by semiquantitative RT-PCR. Rice seedlings were grown in the dark for 3 days and RNA was isolated from the shoots and roots. *Actin1* was used as an internal control.

The *mhz4* coleoptiles showed enhanced ethylene response (Figure 1A, C). We determined whether ABA addition would complement the mutant response to ethylene. Without ABA, the coleoptile length of *mhz4* mutant was significantly longer than that of WT seedlings in the presence or absence of ethylene (Figure 5A and 5B). When 0.1 µM ABA was applied to roots, however, the *mhz4* coleoptiles were identical in length to that of WT seedlings with or without ethylene treatment (Figure 5A and 5B). These observations suggest that application of 0.1 µM ABA substantially restored the *mhz4* coleoptile ethylene response to WT levels and that ABA-deficiency is responsible for the ethylene hypersensitivity of *mhz4* coleoptiles.

Enhanced ethylene response can be caused by ethylene overproduction and/or enhanced signal transduction. We then measured ethylene production in *mhz4*. The *mhz4* mutant produced 2 to 12 times more ethylene than WT and application of 1 µM ABA to etiolated *mhz4* seedlings dramatically reduced the ethylene levels, suggesting that ABA-deficiency in *mhz4* mutant promote ethylene biosynthesis (Figure 5C). Furthermore, quantitative PCR analysis revealed that the ethylene biosynthetic genes *OsACS2*, *OsACS6*, *OsACO3* and *OsACO5* were all elevated in *mhz4* shoots, likely contributing to the enhanced ethylene production (Figure 5D).

We next investigated the contribution of ethylene overproduction to the enhanced ethylene response of *mhz4* coleoptiles through treatment with the ethylene biosynthesis inhibitor 1-aminoethoxyvinyl-glycine (AVG). Application of 5 µM AVG removed most of the increased ethylene production in *mhz4* mutant, but exerted limited effect on the coleoptile ethylene response (Figure 5E and 5F, Figure S2). This result suggests that ethylene overproduction is not the main reason for the enhanced ethylene response in *mhz4* coleoptiles. We further examined the possibility of ethylene signaling and found that *OsEIN2* transcripts were particularly enriched in *mhz4* shoots compared with that of WT (Figure 5G). These results suggest that the enhanced ethylene response of *mhz4* coleoptiles is most likely caused by enhanced ethylene signaling.

### *MHZ4* Overexpression Alters Ethylene Response in Roots and Coleoptiles

**Figure 6:**
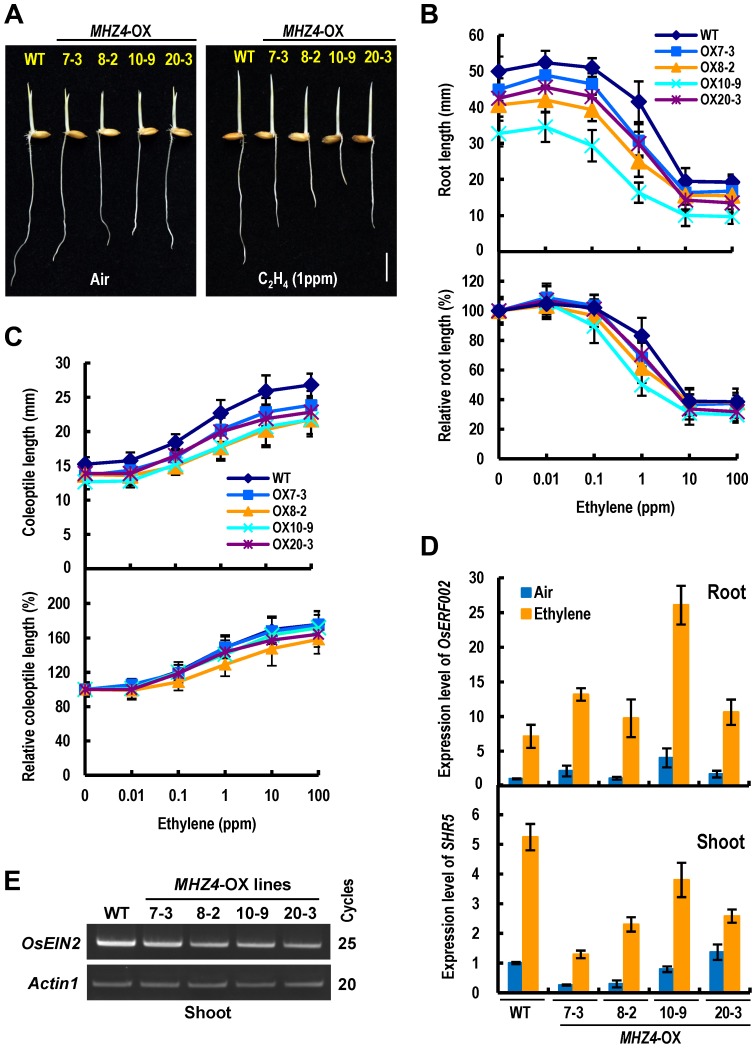
*MHZ4* overexpression confers enhanced and reduced ethylene responses in roots and coleoptiles, respectively. (*A*) Ethylene response phenotypes in WT and *MHZ4-*overexpressing (*MHZ4*-OX) lines. Rice seedlings were grown in dark for 2.5 d in the presence or absence of 1 ppm ethylene. Bar = 10 mm. (*B*) Ethylene dose-response curves for root length (top) and relative root length (bottom) in WT and *MHZ4*-OX lines. (*C*) Ethylene dose-response curves for coleoptile length (top) and relative coleoptile length (bottom) in WT and *MHZ4*-OX lines. Others are as in (B). (*D*) Expressions of ethylene-inducible genes in roots (top) and shoots (bottom) of WT and *MHZ4*-OX lines. Dark-grown 2 d-old seedlings were treated with or without 10 ppm of ethylene for 8 h and the RNA was isolated for quantitative RT-PCR. Data are the mean ± SD of four replicates. (*E*) *OsEIN2* gene expression in shoots of WT and *MHZ4*-OX lines detected by semi-quantitative PCR. Rice seedlings were grown in dark for 3 days and RNA was isolated from shoots. *Actin1* was used as an internal control.

To further study the function of *MHZ4* in rice ethylene response, we transformed the gene into WT rice plants under the control of the CaMV 35S promoter. *MHZ4*-overexpressing (*MHZ4*-OX) lines were identified by semiquantitative RT-PCR (Figure S3), and four representative lines were used for further analysis. The four transgenic lines all showed slightly but significantly shorter roots (*P*<10^−8^) and coleoptiles (*P*<10^−8^) in the dark compared with WT seedlings, indicating constitutive growth inhibition of the roots and coleoptiles in *MHZ4*-OX plants (Figure 6A–6C). Upon ethylene treatment, the *MHZ4*-OX lines exhibited strong inhibition of root growth but less promotion of coleoptile growth in comparison with WT seedlings, indicating enhanced ethylene response in the roots but reduced response in the coleoptiles of *MHZ4*-OX lines (Figure 6A–6C). Examination of the ethylene-inducible gene *OsERF002* in the roots and *SHR5* in the shoots further proved the ethylene responsiveness of *MHZ4*-OX lines (Figure 6D). Additionally, in the shoots, *OsEIN2* transcript levels were down-regulated in *MHZ4*-OX lines compared with that in WT seedlings (Figure 6E), suggesting that the reduced ethylene responsiveness of the coleoptiles is likely related to the reduction of ethylene signaling. Together, these results indicate that *MHZ4* overexpression leads to different ethylene responses in roots and coleoptiles.

### Genetic Interactions of *MHZ4* and Ethylene Signaling Pathway

**Figure 7:**
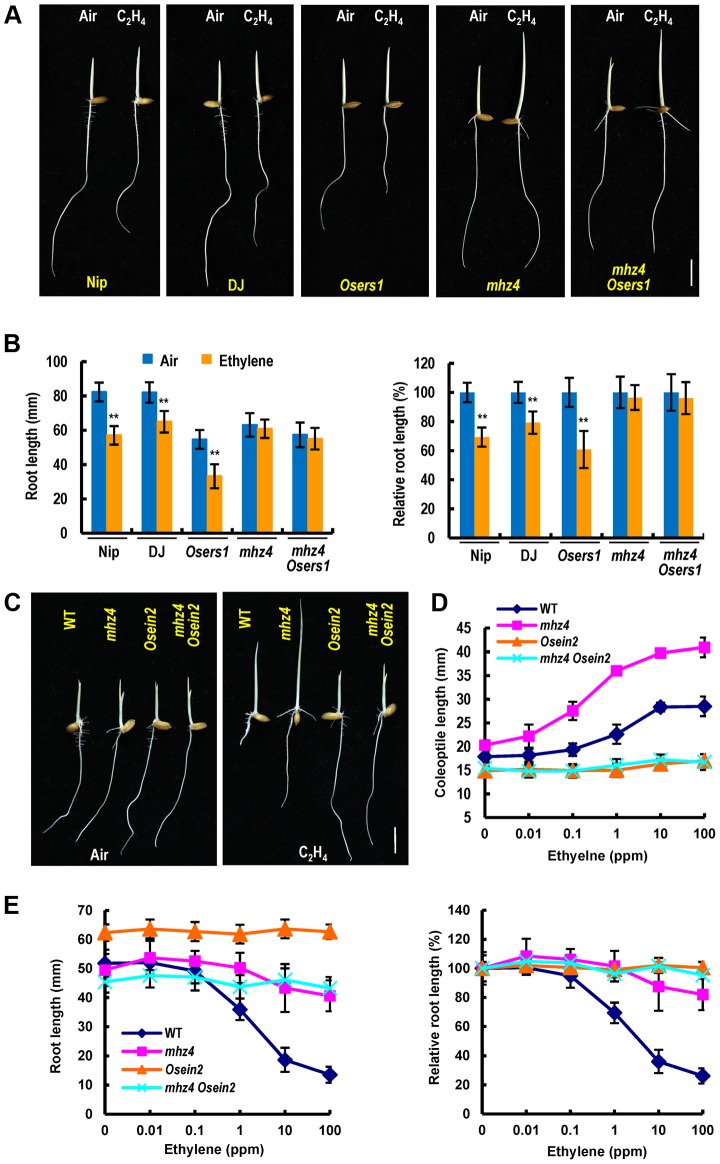
Genetic interactions of *MHZ4* with ethylene receptor gene and *MHZ7/OsEIN2* gene. (*A*) Comparison of ethylene-response phenotypes of *mhz4 Osers1* double mutant and the single mutants. Nipponbare (Nip) and Dongjin (DJ) are wild types. Double mutant between *mhz4* and ethylene receptor *OsERS1* mutant *Osers1* (Dongjin background) were generated by crossing. Rice seedlings were grown in the dark for 3 d in the presence or absence of 1 ppm ethylene. (*B*) Quantification of root length (left) and relative root length (right) of the mutants in (A). Each column is average of 50 seedlings and bars indicate SD. ** indicate significant difference compared to air control at P<0.01. (*C*) Ethylene response phenotypes of double mutant *mhz4 Osein2* and the single mutants. *Osein2/mhz7-1* is in Nipponbare background. Rice seedlings were grown in dark for 3 d in the presence or absence of 10 ppm ethylene. (*D*) Ethylene dose-response curves for coleoptile length in various mutants. Each point is average of 20 seedlings and bars indicate SD. (*E*) Ethylene dose-response curves for root length (left) and relative root length in various mutants (right). Others are as in (D). Bars = 10 mm.

We further examined the genetic relationship of *MHZ4* and ethylene signaling pathway through double mutant analyses. A loss-of function *Osers1* mutant was obtained from the POSTECH Biotech Center [50] and was identified by PCR-based analyses (Figure S4). The roots of *Osers1* etiolated seedlings were significantly shorter than that of WT seedlings in the absence of ethylene and displayed a strong ethylene response phenotype following treatment with 1 ppm ethylene, indicating the presence of enhanced ethylene response in *Osers1* roots (Figure 7A and 7B). The roots of *mhz4 Osers1* double mutant were slightly shorter than that of the *mhz4* single mutant but exhibited ethylene insensitive response that was indistinguishable from that of *mhz4*, suggesting that *OsERS1*-mediated root ethylene response requires *MHZ4* function (Figure 7A and 7B). The coleoptiles of *Osers1* did not exhibit apparent ethylene response phenotype compared to that of WT (Figure 7A).

We constructed *mhz4 Osein2* double mutant to analyze the genetic interaction of *MHZ4* with *OsEIN2* in coleoptile ethylene response. The *Osein2/mhz7-1* is a mutant showing ethylene insensitivity in both roots and coleoptiles previously identified [45]. The coleoptiles of *mhz4 Osein2* double mutant showed complete ethylene insensitivity similar to that of *Osein2* seedlings but unlike the *mhz4* coleoptiles, indicating that *mhz4* requires *OsEIN2* in regulation of coleoptile ethylene response (Figure 7C and 7D). On the other hand, the roots of *mhz4 Osein2* double mutant showed complete ethylene insensitivity similar to the case in *Osein2* roots (Figure 7E). Considering that *mhz4* roots still had some ethylene response and this weak response was completely abolished in the double mutant, we propose that the residual ethylene response observed in *mhz4* roots was dependent on OsEIN2 function.

### Effects of *MHZ4* on Plant Growth and Yield-Related Traits

**Figure 8:**
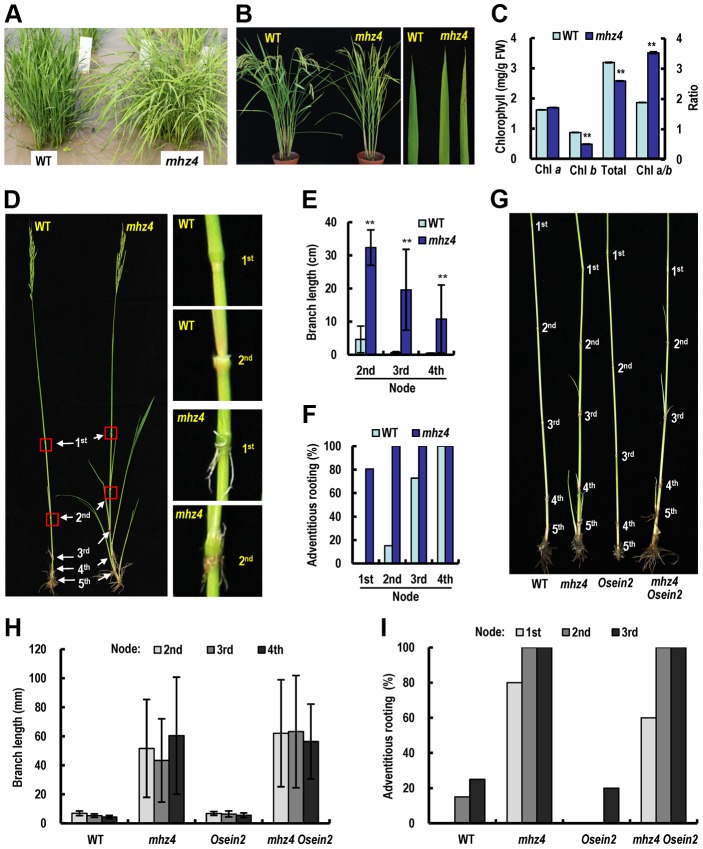
Phenotypic comparison of field-grown plants. (*A*) Phenotypes of Five-week-old seedlings of WT and *mhz4*. (*B*) Plant phenotypes after heading. Please note the spots on some *mhz4* leaves but not WT leaves. (*C*) Chlorophyll contents in the fourth leaves of plants. Chlorophyll was extracted with 95% ethanol from the fourth leaves from the top at heading stage. Each column is average of three measurements and bars indicate SD. ‘**’ indicate significant difference compared to WT (P<0.01). (*D*) Formation of branches and nodal adventitious roots in the main tiller of WT and *mhz4* plants. (*E*) Branch length at each node from the main tillers of WT and *mhz4*. Each column is average of 30 to 35 plants and bars indicate SD. ‘**’ indicate significant difference compared to WT (P<0.01). (*F*) Percentage of nodal adventitious roots in the main tillers. 30 to 35 plants were investigated for WT and *mhz4*. (*G*) Effects of *OsEIN2* mutation on branching and adventitious root formation of *mhz4*. Representative plants of WT, *mhz4*, *Osein2* and *mhz4 Osein2* double mutant were compared. (*H*) Quantification of branch length at each node in main tillers from 10–20 plants in (G). (*I*) Percentage of adventitious root formation at each node in main tillers from 10–20 plants in (G).

We compared phenotypes of field-grown *mhz4* mutant, *MHZ4*-OX lines and WT plants. At seedling stage, the *mhz4* mutant had flowing leaves with yellow green color compared with WT plants (Figure 8A). From vegetative to reproductive stages, the leaves of *mhz4* appeared to be pale green and some leaves had brown dots on the leaf tip (Figure 8B). Chlorophyll (Chl) analysis revealed that *mhz4* had a reduction in Chl *b* and total Chl contents and an increase in Chl a/*b* ratio compared with WT leaves (Figure 8C), indicating that *mhz4* mutation interferes with Chl *b* biosynthesis. This may alter the light harvesting antenna size, thus affecting photosynthetic efficiency [51], [52]. For flowering time, both *mhz4* mutant and the four *MHZ4*-OX lines exhibited delayed heading time in comparison with WT plants (Table S2).

At maturation stage, *mhz4* mutant exhibited dramatic growth of branches developed on upper nodes and adventitious roots formation on the nodes in comparison with WT plants (Figure 8D–8F). Similar phenotype was observed in the *mhz4 Osein2* double mutant in different years (Figure 8G–8I; Figure S5). These results suggest that *MHZ4* negatively regulate axillary bud growth and adventitious rooting possibly through ABA function but not ethylene signaling.

Plant height is an important agronomic trait associated with rice yield. Ethylene positively regulates rice plant height by promoting stem elongation [53]. The *mhz4* plants were taller than WT and all the four *MHZ4*-OX lines were shorter than WT plants (Table S2). The *mhz4 Osein2* double mutant was shorter than *mhz4* plants but was similar to that of *Osein2* plants (Table S2). These observations suggest that *MHZ4* negatively regulate plant height in rice in an *OsEIN2*-dependent manner. The promoted plant height of *mhz4* is possibly due to the increased ethylene production (Figure 5C). The numbers of effective tiller (tillers producing panicles with at least five filled grains) in WT and *mhz4* were identical, whereas two of the four *MHZ4*-OX lines produced more effective tillers than the WT (Table S2). Panicle length of *mhz4* mutant was shorter than that of WT (Table S2).

Grain-related traits were also examined. The *mhz4* grains had no dormancy and displayed preharvest sprouting phenotype (Figure S6A and S6B). This is a typical characteristic for ABA-deficient mutants of rice [54]. Seed-setting rate and 1000-grain weight were all significantly reduced in *mhz4* mutant (Table S2). The grain shape of WT and *mhz4* was further examined. The *mhz4* mutant showed a decrease in grain thickness and an increase in grain length, implying that *MHZ4* mutation affects grain shape (Figure S6C). We further performed a time course analysis of grain dry weight during grain filling and found that the grain weight of *mhz4* increased more slowly than that of WT, suggesting that grain filling is hindered in *mhz4* mutant (Figure S6D). These results indicate that alteration of *MHZ4* expression affects branching, plant height and grain-related traits.

## Discussion

We characterized a rice ethylene-response mutant *mhz4*, which showed reduced ethylene response in roots but enhanced ethylene response in coleoptiles. *MHZ4* encoded a chloroplast-localized membrane protein homologous to *Arabidopsis* ABA4 in ABA biosynthesis pathway. *MHZ4* mutation reduced ABA level but enhanced ethylene production. *MHZ4* overexpression enhanced ethylene responses in roots but reduced ethylene response in coleoptiles. Genetically, *MHZ4* acts at or downstream of the ethylene receptors to positively regulate root ethylene response likely through ethylene-triggered ABA accumulation. Additionally, *MHZ4* acts at or upstream of *OsEIN2* to negatively regulate coleoptile ethylene response possibly through modulating *OsEIN2* expression. *MHZ4* also affects agronomic traits. Our findings reveal a novel mode of interplay between ethylene and ABA.

The roots of *mhz4* seedlings displayed about 20% growth inhibition (80% insensitivity) when treated with 100 ppm ethylene (Figure 1B). By contrast, the WT roots exhibited about 70% inhibition under the same concentration of ethylene. This comparison reveals that *MHZ4* mediates a large part of the ethylene-inhibition of root growth. Since *MHZ4* mutation reduced ABA contents possibly through disruption of a branch of ABA biosynthesis (Figure 4A), we propose that ABA may be the factor participating in ethylene-inhibition of root growth. Five pieces of evidence supports this conclusion. (1) Exogenous application of ABA largely recovered the defective response of *mhz4* roots to ethylene; (2) *MHZ4*-overexpression resulted in enhanced ethylene response in roots; (3) Ethylene induced *MHZ4* expression and ABA accumulation particularly in roots; (4) Ethylene-induced ABA mediates expression of some ethylene-responsive genes; (5) The ethylene receptor *OsERS1*-mediated root ethylene response required *MHZ4* function. The present conclusion that ethylene inhibited root growth largely/partly through ABA function is in contrast to that observed in *Arabidopsis*, where ethylene-mediated root inhibition is independent of ABA action [21], [22], [26]. This difference indicates that rice, as a monocot or a semiaquatic plant, may have adopted a novel mechanism for ethylene-ABA interaction.

We have identified another rice ABA-deficient mutant *mhz5* that showed a similar ethylene response phenotype as *mhz4* and the corresponding gene *OsCRTISO* encodes the carotenoid isomerase that acts at early step in ABA biosynthetic pathway (Yin et al., unpublished). Consequently, it seems that the ABA-dependent ethylene effect on rice root growth is not restricted to *MHZ4*, possibly other ABA biosynthetic genes and even the signaling component genes may affect the root ethylene response in rice.

In *Arabidopsis*, extensive studies have established that ethylene inhibits root growth through auxin action via modulating its biosynthesis, transport and/or signaling [55]–[62]. Auxin response occurring in the elongation zone mediates a substantial part of ethylene effect on root growth [63]. In monocotyledonous plant *Brachypodium*, ethylene may inhibit auxin biosynthesis in IPA pathway to control root elongation, which is different from the case in *Arabidopsis* [64]. In rice, our present results demonstrate that ethylene inhibits root growth largely through ABA function. Previous studies also discover that ethylene triggers ABA biosynthesis, leading to growth inhibition [65]. It is unknown whether auxin is also involved in this process. ABA may act through auxin pathway or vice versa, or the two hormones might act independently to mediate ethylene response. Further investigation toward the relationship among these hormones should shed light on their complicated interaction in control of rice roots.

*MHZ4* mutation drastically enhances ethylene emission possibly through activation of ethylene biosynthesis genes, and ABA inhibits ethylene production (Figure 5C). Considering that ethylene induces ABA to inhibit root growth (Figure 4), the ABA suppression of ethylene generation may represent a negative feedback control mechanism to alleviate ethylene effects on roots.

The coleoptiles of *mhz4* were slightly but significantly longer than that of WT seedlings (Figure 5B), indicating that ABA acts as an inhibitor of rice coleoptile elongation. Similar effect of ABA was observed in rice seedlings treated with fluridone, an inhibitor of ABA biosynthesis, which reduced the levels of endogenous ABA in coleoptiles and in turn promoted coleoptile growth [66]. In contrast to the ABA effect, ethylene stimulates coleoptile growth in rice (Figure 1; [46]), suggesting that the two hormones have antagonistic interaction. That *MHZ4* mutation enhanced ethylene production and ethylene response whereas its overexpression reduced ethylene response in coleoptiles (Figure 1, 5, and 6) further supports the antagonistic relationship of ABA and ethylene. In *Arabidopsis*, ABA biosynthesis gene *ABA2* mutation also resulted in elevated ethylene production [67]; however, ethylene response of this mutant was not altered [26], [67], indicating presence of conserved and diverged aspects in interactions of ABA and ethylene.

In WT shoots, ethylene slightly inhibits ABA levels and may hence promote shoot/coleoptile elongation (Figure 4A). In *mhz4* shoots, the ABA level is slightly induced by ethylene but the induction is not statistically significant (P = 0.089168). Additionally, the ABA level in *mhz4* shoots is quite low (about 1/10 of the WT level) compared with that in WT. The small increase in ABA contents in *mhz4* shoots after ethylene treatment may be not enough to affect its ethylene response. However, we could not exclude the possibility that ethylene-altered ABA levels may have some subtle effects on ethylene responses.

Although elevated ethylene production was observed in *mhz4* mutant, AVG treatments that inhibited ethylene production did not significantly altered ethylene response (Figure 5E), suggesting that the enhanced ethylene response in *mhz4* coleoptiles is likely caused by enhanced ethylene signaling rather than ethylene overproduction. In support of this prediction, transcript levels of the central ethylene signaling component *OsEIN2* were up-regulated in *mhz4* shoots (Figure 5G). Further genetic analysis revealed that *mhz4* ethylene-response phenotypes in coleoptiles required *OsEIN2* function. Collectively, *MHZ4*-dependent ABA pathway negatively regulates rice coleoptile ethylene response at least in part through modulating *OsEIN2* expression. It should be mentioned that although the ethylene overproduction in *mhz4* mutant did not play major roles in enhanced ethylene response, the basal level of ethylene did contribute to the coleoptile elongation because AVG reduced the coleoptile length of *mhz4* to the WT level (Figure 5E).

Our present results reveal that ABA and ethylene are synergistic in root growth inhibition but antagonistic in coleoptile elongation. The opposite effects of *mhz4* mutation on ethylene response of rice root and coleoptile may be due to alterations of expressions of ethylene-responsive genes in these organs (Figure 1D, E). Previous studies have reported antagonistic interactions of ethylene and ABA in several processes such as seed germination, stomatal closure and submerge-induced shoot elongation of semiaquatic plants, and synergistic interactions in tomato fruit ripening and abiotic stress responses [68]. These findings imply that ethylene-ABA interplay is quite complicated, depending on biological processes, tissue/organs, and plant species. Why plants have these different interactions remains unclear. It is possible that multiple interaction manners allow plants to be more adapted to the changing environments at different growth and developmental stages.

At mature stage, *mhz4* plants had branches and adventitious roots at nodes of higher positions (Figure 8). This phenomenon is rarely observed in rice and not dependent on OsEIN2 function of ethylene signalling through double mutant analysis, suggesting that ABA itself inhibits branching and formation of adventitious root. This function probably represents a novel aspect of ABA roles during rice development. It has been reported that a higher ratio of ABA to ethylene in rice spikelets is required to maintain a faster grain-filling rate [69]. In our *mhz4* mutant, a low ABA level but a high ethylene production was noted and the low ratio of ABA to ethylene may be related to the impaired grain filling (Figure S6). However, in *MHZ4*-overexpressing plants, grain-related traits were not widely improved (Table S2), implying that these traits are likely regulated in a complicated manner.

The *MHZ4* seems to have a negative role on plant height because the *mhz4* mature plants are taller than WT whereas the overexpressing lines are shorter than WT (Table S2). *MHZ4* mutation activated ethylene production which increased plant height through *OsEIN2* function. Our results are similar with those obtained in *Arabidopsis* and tomato, where ABA-deficient mutants exhibit stunted shoot growth due to increased ethylene production [67], [70]. It should be noted that ethylene promotes shoot growth in rice but inhibits this process in *Arabidopsis* and tomato.

In conclusion, we demonstrate that ethylene inhibits rice root growth through ABA action, and ABA negatively regulates coleoptile ethylene response through modulating *OsEIN2* expression. Synergistic interaction in roots but antagonistic interaction in coleoptiles was revealed between ABA and ethylene, providing new insights into understanding of their complicated interplay.

## Materials and Methods

### Plant Materials and Growth Conditions

The *mhz4* and *Osein2*/*mhz7-1* mutants were identified previously [45]. The *OsERS1* (LOC_Os03g49500) T-DNA knockout mutant *Osers1* (PFG_1B-08531.L) is in Dongjin (DJ) background and was obtained from the POSTECH Biotech Center [50]. The homogenous *Osers1* mutation was identified by PCR using the T-DNA left border primer PR152 (5′-TTGGTTAGAGAACAGCACAA-3′) and gene-specific primers flanking the insertion site: PR139 (5′-AATAAATGATTGGCCAGAGC-3′) and PR140 (5′-TGCTTCTCAGTATCCTTTGT-3′). The *Osein2/mhz7-1* harbors a 24-bp deletion in the cording region of *OsEIN2* (LOC_Os07g06130), and the mutation was identified as previously described [45]. For ethylene treatment, rice seedlings were grown on a stainless steel sieve that was placed in an air-tight plastic box with various concentrations of ethylene [45]. The seedlings were incubated at 28°C in the dark for 2 to 3 days as indicated in each experiment. For ABA or NDGA treatment, rice seedlings were grown on a stainless steel sieve and various concentrations of ABA (Sigma, A1049) or 100 µM NDGA (Sigma) was added to the water. The stock solutions of ABA and NDGA were prepared in ethanol. For AVG treatment, rice seedlings were grown on eight layers of cheesecloth saturated with 5 µM AVG (Sigma) in Petri dishes. For field experiments, rice plants were grown in the Experimental farm Station of the Institute of Genetics and Developmental Biology in Beijing from May to October of each year.

### Map-Based Cloning of *MHZ4* Gene

F2 mapping populations were generated from crosses between *mhz4* mutant and indica variety Minghui 63. Genomic DNA was isolated from etiolated seedlings with mutant phenotype. For primary mapping, bulked segregant analysis (BSA) was performed using a DNA pool from 15 mutant individuals selected from the F2 population between *mhz4* and MH63. The markers used in BSA included 30 published SSR markers (http://www.gramene.org) and 117 insertion-deletion (Idl) markers. A total of 480 mutant individuals selected from the F2 populations were used for fine mapping. *MHZ4* locus was mapped to chromosome 1 between Idl1–1.55 and (5′- CAGGGCAATCTGTCAAAGCT-3′ and 5′- CTAAAGATCAGTACTGGGCAC-3′) and Idl1–1.58 (5′- CTCTTTGTCAAGCTTATTTACC-3′ and 5′- ACAGATCCGTATGTTTATAGTG-3′) in the region of 1.55 Mb to 1.58 Mb (Genebank accession number: NC_008400), which contains 4 genes. The candidate gene was finally determined by DNA sequencing of all the candidate genes within this region.

To confirm the 18-bp deletion in the genomic DNA of *mhz4*, DNA fragment length polymorphism between wild-type (WT) and the mutant was examined using PCR with primers (5′- CTCTGTTCCGGCCTCGCGCA -3′ and 5′- AGGACGGCGATGGTGCCCCA -3′). The 81-bp insertion in the *mhz4* cDNA was detected by PCR amplification of the full-length cDNA using primers (5′- ATGGCGGCTCTCCTCCTCCT -3′ and 5′-TCAATGTGAGCGACCAATTGAAC-3′).

### Quantitative PCR and RNA-Seq Analyses

Total RNAs were extracted from various rice tissues using TRIZOL reagent (Invitrogen) according to the manufacturer's instructions. Quantitative PCR and/or semi-quantitative PCR were carried out as described previously [45]. The primers are listed in Table S1. For RNA-seq analysis, two-day-old etiolated seedlings of WT and *mhz4* mutant were treated with air or 10 ppm ethylene for 8 h. The libraries were prepared from 10 µg total RNA using NEBNext Ultra RNA Library Prep Kit (NEB, USA), and each sample contains two biological replicates. Clean reads were mapped to rice MSU7.0 genome using TopHat, and analyzed using Cufflinks as described [71].

### Transient Expression Assay

To generate *MHZ4-GFP* fusion construct, the MHZ4 coding sequence was PCR amplified (for primers see Table S1), digested with *Xhol*I/*Nco*I and fused in frame to the 5′-end of *GFP* in a pUC18-based vector under the control of CaMV35S promoter. The fusion gene was transiently expressed in tobacco leaves by microprojectile bombardment using a Bio-Rad PDS-1000/He particle delivery system [9]. The images were taken using a confocal microscope (Leica TCS SP5). Excitation/emission wavelengths were set at 488 nm/500–530 nm for GFP fluorescence and at 570 nm/640 nm for chlorophyll autofluorescence.

### Generation of Transgenic Rice Plants

To generate *MHZ4pro::GUS* construct, a 2048-bp promoter region upstream of the start codon was PCR amplified and cloned into *Sse8387*I/*BamH*I sites of pCAMBIA2300-35S-GUS vector to replace the CaMV35S promoter. To generate *MHZ4*-complementation construct, the genomic DNA (4213 bp) of *MHZ4* was PCR amplified and subcloned into *Sse83871*I/*Sal*I-digested pCAMBIA2300-35S vector. To generate the *35S::MHZ4* construct, the *MHZ4* coding sequence was PCR amplified and inserted into *BamH*I/*Sal*I-digested pCAMBIA2300-35S vector. The primers used for these constructions are listed in Table S1. The constructs were transfected into *Agrobacterium tumefaciens* strain EHA105 by electroporation. Rice transformation was performed as previously described [42]. The *MHZ4pro*::*GUS* or *35S::MHZ4* construct was transformed into rice variety Nipponbare. The *MHZ4*-complementation construct was transferred into *mhz4* mutant. Positive transgenic plants were confirmed by PCR using *NPT II* gene-specific primers (Table S1). Homozygous transgenic lines were selected using Kanamycin treatment (50 mg/L).

### GUS Staining

Rice seedlings or organs were fixed in 90% acetone on ice for 15 min, rinsed with staining buffer (100 mM Na_3_PO_4_ buffer pH 7.0, 10 mM EDTA, 5 mM potassium ferricyanide, 5 mM potassium ferrocyanide, 0.1% Triton X-100), and vacuum infiltrated with staining solution (staining buffer containing 0.5 mg/ml X-Gluc (Sigma, B8049) for 15 minutes. The samples were incubated at 37°C in the dark. After staining, green tissues were decolorized in 70% ethanol and then in ethanol/acetic acid (6∶1) until the chlorophyll was removed. The samples were observed using stereo microscopy (Leica, M165 FC).

### Measurement of Ethylene and ABA

Ethylene production of WT and *mhz4* mutant in 4 d-old etiolated seedlings, two-week-old green seedlings, mature leaves and panicles was determined by gas chromatography (GC2014, Shimadzu, Japan) equipped with a flame ionization detector. ABA contents in WT and *mhz4* were detected using 4 d-old etiolated seedlings or 2 d-old etiolated seedlings treated with or without 10 ppm of ethylene for 48 h. Plant tissues (200 mg) were ground into powder in liquid nitrogen. ABA was determined by UPLC-MS/MS (UPLC-Quattro Premier XE) at the National Center of Plant Gene Research (IGDB, CAS, Bejing, China) as described [72]. For both ethylene and ABA detection, each material or treatment includes three duplications.

### Measurements of Agronomic Traits in Field-Grown Plants

Heading date of WT, *mhz4* mutants and *MHZ4*-OX lines was recorded when the first spike of a rice plant emerged about 1 cm above the sheaths. Twenty to thirty individuals were examined for each material. After harvest, 20 plants from each material were used for measurements of agronomic traits as previously described [45].

## Supporting Information

Figure S1 ABA dose-response curves for root and coleoptile growth in WT. Rice seedlings were grown in the dark for 3 d in the presence of various concentrations of ABA. Each point is average of 35 to 40 seedlings and bars indicate SD.
(TIF)

Figure S2 Effects of AVG treatment on ethylene production and coleoptile ethylene response of *mhz4* and WT seedlings. (*A*) Ethylene production in the presence of various concentrations of AVG. Data are the mean ± SD of three replicates. (*B*) Coleoptile phenotypes of the seedlings grown in the dark for 2.5 days in the presence or absence of 10 ppm ethylene, supplemented with or without 5 µM AVG.
(TIF)

Figure S3 *MHZ4 g*ene expression levels in overexpressing lines detected using semiquantitative RT-PCR. *Actin1* was used as a control.
(TIF)

Figure S4 Identification of the *Osers1* mutant. (*A*) Schematic representation of *OsERS1* (LOC_Os03g49500) gene structure. The T-DNA insertion site and the primer positions are indicated. (*B*) PCR genotyping for the *Osers1* mutant and WT (Dongjin). (*C*) *OsERS1* expression in *Osers1* and WT detected by RT-PCR analysis with amplification of the full-length cDNA. *Actin1* was used as an internal control.
(TIF)

Figure S5 Quantification of branching and adventitious rooting in WT, *mhz4*, *Osein2* and *mhz4 Osein2* double mutant plants in a different year. (*A*) Branch length at each node in main tillers from 20 plants. (*B*) Percentage of adventitious root formation at each node in main tillers in 20 plants in (A).
(TIF)

Figure S6 Preharvest sprouting and grain-related traits of *mhz4* mutant. (*A*) Preharvest sprouting of *mhz4*. Arrow heads indicate germinated seeds on a panicle. (*B*) Germination rates of freshly harvested seeds of WT and *mhz4*. Each point is the percentage of germinated seeds among 100 seeds. (*C*) Comparison of grain thickness (top left panel), grain length (bottom panel) and their quantification results (top right panel) in WT and *mhz4*. Each column is an average of 100 grains and bars indicate SD. ‘**’ indicate significant difference compared to WT (P<0.01). (*D*) Time-course of grain-filling after fertilization. Each point is an average of 50–100 grains and bars indicate SD.
(TIF)

Table S1 Primers used gene expression analysis and plasmid construction.
(DOCX)

Table S2 Yield-related traits in WT, *mhz4* mutant and *MHZ4*-overexpressing lines. Each value is average of 20 to 24 individuals. * and ** indicate significant difference compared to WT at P<0.05 and P<0.01, respectively. Data from two years are presented.
(DOCX)

## References

[1] Abeles FB, Morgan PW, Saltveit JME (1992) Ethylene in Plant Biology. 2nd ed. (San Diego, CA: Academic Press).

[2] Bleecker AB, Kende H (2000) Ethylene: a gaseous signal molecule in plants. Annu Rev Cell Dev Biol 16: 1–18. 11031228 10.1146/annurev.cellbio.16.1.1

[3] Hall B, Shakeel S, Schaller GE (2007) Ethylene receptors: ethylene perception and signal transduction. J Plant Growth Regul 26: 118–130.

[4] Ju C, Chang C (2012) Advances in ethylene signalling: protein complexes at the endoplasmic reticulum membrane. AoB PLANTS 2012: pls031 doi:10.1093/aobpla/pls031 23119138 10.1093/aobpla/pls031 PMC3485614

[5] Kendrick MD, Chang C (2008) Ethylene signaling: new levels of complexity and regulation. Curr Opin Plant Biol 11: 479–485. 18692429 10.1016/j.pbi.2008.06.011 PMC2562597

[6] Shakeel SN, Wang X, Binder BM, Schaller GE (2013) Mechanisms of signal transduction by ethylene: overlapping and nonoverlapping signalling roles in a receptor family. AoB PLANTS 5: plt010 doi:10.1093/aobpla/plt010 23543258 10.1093/aobpla/plt010 PMC3611092

[7] Chen YF, Randlett MD, Findell JL, Schaller GE (2002) Localization of the ethylene receptor ETR1 to the endoplasmic reticulum of *Arabidopsis* . J Biol Chem 277: 19861–19866. 11916973 10.1074/jbc.M201286200

[8] Gao ZY, Chen YF, Randlett MD, Zhao XC, Findell JL, et al (2003) Localization of the Raf-like kinase CTR1 to the endoplasmic reticulum of *Arabidopsis* through participation in ethylene receptor signaling complexes. J Biol Chem 278: 34725–34732. 12821658 10.1074/jbc.M305548200

[9] Ma B, Cui ML, Sun HJ, Takada K, Mori H, et al (2006) Subcellular localization and membrane topology of the melon ethylene receptor CmERS1. Plant Physiol 141: 587–597. 16617090 10.1104/pp.106.080523 PMC1475473

[10] Bisson MMA, Bleckmann A, Allekotte S, Groth G (2009) EIN2, the central regulator of ethylene signalling, is localized at the ER membrane where it interacts with the ethylene receptor ETR1. Biochem J 424: 1–6. 19769567 10.1042/BJ20091102

[11] Qiao H, Chang KN, Yazaki J, Ecker JR (2009) Interplay between ethylene, ETP1/ETP2 F-box proteins, and degradation of EIN2 triggers ethylene responses in *Arabidopsis* . Genes Dev 23: 512–521. 19196655 10.1101/gad.1765709 PMC2648649

[12] Ju C, Yoon GM, Shemansky JM, Lin DY, Ying ZI, et al (2012) CTR1 phosphorylates the central regulator EIN2 to control ethylene hormone signaling from the ER membrane to the nucleus in *Arabidopsis* . Proc Natl Acad Sci U S A 109: 19486–19491. 23132950 10.1073/pnas.1214848109 PMC3511113

[13] Kamiyoshihara Y, Tieman DM, Huber DJ, Klee HJ (2012) Ligand-induced alterations in the phosphorylation state of ethylene receptors in tomato fruit. Plant Physiol 160: 488–497. 22797658 10.1104/pp.112.202820 PMC3440222

[14] Qiao H, Shen Z, Huang SS, Schmitz RJ, Urich MA, et al (2012) Processing and subcellular trafficking of ER-tethered EIN2 control response to ethylene gas. Science 338: 390–393. 22936567 10.1126/science.1225974 PMC3523706

[15] Wen X, Zhang C, Ji Y, Zhao Q, He W, et al (2012) Activation of ethylene signaling is mediated by nuclear translocation of the cleaved EIN2 carboxyl terminus. Cell Res 22: 1613–1616. 23070300 10.1038/cr.2012.145 PMC3494400

[16] Guo HW, Ecker JR (2003) Plant responses to ethylene gas are mediated by SCF (EBF1/EBF2)-dependent proteolysis of EIN3 transcription factor. Cell 115: 667–677. 14675532 10.1016/s0092-8674(03)00969-3

[17] Potuschak T, Lechner E, Parmentier Y, Yanagisawa S, Grava S, et al (2003) EIN3-dependent regulation of plant ethylene hormone signaling by two *Arabidopsis* F box proteins: EBF1 and EBF2. Cell 115: 679–689. 14675533 10.1016/s0092-8674(03)00968-1

[18] Vandenbussche F, Van Der Straeten D (2007) One for all and all for one: cross-talk of multiple signals controlling the plant phenotype. J Plant Growth Regul 26: 178–187.

[19] Yoo SD, Cho Y, Sheen J (2009) Emerging connections in the ethylene signalling network. Trends Plant Sci 14: 270–279. 19375376 10.1016/j.tplants.2009.02.007 PMC3063992

[20] Zhao Q, Guo H (2011) Paradigms and paradox in the ethylene signaling pathway and interaction network. Mol Plant 4: 626–634. 21690206 10.1093/mp/ssr042

[21] Beaudoin N, Serizet C, Gosti F, Giraudat J (2000) Interactions between abscisic acid and ethylene signaling cascades. Plant Cell 12: 1103–1115. 10899977 10.1105/tpc.12.7.1103 PMC149052

[22] Ghassemian M, Nambara E, Cutler S, Kawaide Y, Kamiya Y, et al (2000) Regulation of abscisic acid signaling by the ethylene response pathway in *Arabidopsis* . Plant Cell 12: 1117–1126. 10899978 10.1105/tpc.12.7.1117 PMC149053

[23] Benschop JJ, Jackson MB, Guhl K, Vreeburg RAM, Croker SJ, et al (2005) Contrasting interactions between ethylene and abscisic acid in *Rumex* species differing in submergence tolerance. Plant J 44: 756–768. 16297068 10.1111/j.1365-313X.2005.02563.x

[24] Benschop JJ, Millenaar FF, Smeets ME, van Zanten M, Voesenek LA, et al (2007) Abscisic acid antagonizes ethylene-induced hyponastic growth in *Arabidopsis* . Plant Physiol 143: 1013–1023. 17158582 10.1104/pp.106.092700 PMC1803718

[25] Wang Y, Liu C, Li K, Sun F, Hu H, et al (2007) *Arabidopsis EIN2* modulates stress response through abscisic acid response pathway. Plant Mol Biol 64: 633–644. 17533512 10.1007/s11103-007-9182-7

[26] Cheng WH, Chiang MH, Hwang SG, Lin PC (2009) Antagonism between abscisic acid and ethylene in *Arabidopsis* acts in parallel with the reciprocal regulation of their metabolism and signaling pathways. Plant Mol Biol 71: 61–80. 19513806 10.1007/s11103-009-9509-7 PMC2716446

[27] Dong H, Zhen Z, Peng J, Chang L, Gong Q, et al (2011) Loss of *ACS7* confers abiotic stress tolerance by modulating ABA sensitivity and accumulation in *Arabidopsis* . J Exp Bot 62: 4875–4887. 21765163 10.1093/jxb/err143 PMC3193000

[28] Li Z, Zhang L, Yu Y, Quan R, Zhang Z, et al (2011) The ethylene response factor AtERF11 that is transcriptionally modulated by the bZIP transcription factor HY5 is a crucial repressor for ethylene biosynthesis in *Arabidopsis* . Plant J 68: 88–99. 21645149 10.1111/j.1365-313X.2011.04670.x

[29] Chen L, Dodd IC, Davies WJ, Wilkinson S (2013) Ethylene limits abscisic acid- or soil drying-induced stomatal closure in aged wheat leaves. Plant Cell Environ 36: 1850–1859. 23488478 10.1111/pce.12094

[30] Nambara E, Marion-Poll A (2005) Abscisic acid biosynthesis and catabolism. Annu Rev Plant Biol 56: 165–185. 15862093 10.1146/annurev.arplant.56.032604.144046

[31] Rock CD, Zeevaart JA (1991) The aba mutant of *Arabidopsis thaliana* is impaired in epoxy-carotenoid biosynthesis. Proc Natl Acad Sci U S A 88: 7496–7499. 11607209 10.1073/pnas.88.17.7496 PMC52327

[32] North HM, Almeida AD, Boutin JP, Frey A, To A, et al (2007) The *Arabidopsis* ABA-deficient mutant *aba4* demonstrates that the major route for stress-induced ABA accumulation is via neoxanthin isomers. Plant J 50: 810–824. 17470058 10.1111/j.1365-313X.2007.03094.x

[33] Neuman H, Galpaz N, Cunningham FX Jr, Zamir D, Hirschberg J (2014) The tomato mutation *nxd1* reveals a gene necessary for neoxanthin biosynthesis and demonstrates that violaxanthin is a sufficient precursor for abscisic acid biosynthesis. Plant J 78: 80–93. 24506237 10.1111/tpj.12451

[34] Iuchi S, Kobayashi M, Taji T, Naramoto M, Seki M, et al (2001) Regulation of drought tolerance by gene manipulation of 9-cis-epoxycarotenoid dioxygenase, a key enzyme in abscisic acid biosynthesis in *Arabidopsis* . Plant J 27: 325–33. 11532178 10.1046/j.1365-313x.2001.01096.x

[35] Cheng WH, Endo A, Zhou L, Penney J, Chen HC, et al (2002) A unique short-chain dehydrogenase/reductase in *Arabidopsis* glucose signaling and abscisic acid biosynthesis and functions. Plant Cell 14: 2723–43. 12417697 10.1105/tpc.006494 PMC152723

[36] Seo M, Peeters AJ, Koiwai H, Oritani T, Marion-Poll A, et al (2000) The *Arabidopsis aldehyde oxidase 3* (*AAO3*) gene product catalyzes the final step in abscisic acid biosynthesis in leaves. Proc Natl Acad Sci U S A 97: 12908–12913. 11050171 10.1073/pnas.220426197 PMC18863

[37] Cutler SR, Rodriguez PL, Finkelstein RR, Abrams SR (2010) Abscisic acid: emergence of a core signaling network. Annu Rev Plant Biol 61: 651–679. 20192755 10.1146/annurev-arplant-042809-112122

[38] Joshi-Saha A, Valon C, Leung J (2011) Abscisic acid signal off the STARting block. Mol Plant 4: 562–580. 21746700 10.1093/mp/ssr055

[39] Ben-Ari G (2012) The ABA signal transduction mechanism in commercial crops: learning from *Arabidopsis* . Plant Cell Rep 31: 1357–1369. 22660953 10.1007/s00299-012-1292-2

[40] Jun SH, Han MJ, Lee S, Seo YS, Kim WT, et al (2004) OsEIN2 is a positive component in ethylene signaling in rice. Plant Cell Physiol 45: 281–289. 15047876 10.1093/pcp/pch033

[41] Yau CP, Wang L, Yu M, Zee SY, Yip WK (2004) Differential expression of three genes encoding an ethylene receptor in rice during development, and in response to indole-3-acetic acid and silver ions. J Exp Bot 55: 547–556. 14754915 10.1093/jxb/erh055

[42] Wuriyanghan H, Zhang B, Cao WH, Ma B, Lei G, et al (2009) The ethylene receptor ETR2 delays floral transition and affects starch accumulations in rice. Plant Cell 21: 1473–1494. 19417056 10.1105/tpc.108.065391 PMC2700534

[43] Zhang W, Zhou X, Wen CK (2012) Modulation of ethylene responses by *OsRTH1* overexpression reveals the biological significance of ethylene in rice seedling growth and development. J Exp Bot 63: 4151–4164. 22451723 10.1093/jxb/ers098 PMC3398448

[44] Wang Q, Zhang W, Yin Z, Wen CK (2013) Rice CONSTITUTIVE TRIPLE-RESPONSE2 is involved in the ethylene-receptor signalling and regulation of various aspects of rice growth and development. J Exp Bot 64: 4863–4875. 24006427 10.1093/jxb/ert272 PMC3830475

[45] Ma B, He SJ, Duan KX, Yin CC, Chen H, et al (2013) Identification of rice ethylene-response mutants and characterization of *MHZ7/OsEIN2* in distinct ethylene response and yield trait regulation. Mol Plant 6: 1830–1848. 23718947 10.1093/mp/sst087

[46] Ku HS, Suge H, Rappaport L, Pratt HK (1970) Stimulation of rice coleoptile growth by ethylene. Planta 90: 333–339. 24499944 10.1007/BF00386385

[47] Joyard J, Ferro M, Masselon C, Seigneurin-Berny D, Salvi D, et al (2009) Chloroplast proteomics and the compartmentation of plastidial isoprenoid biosynthetic pathways. Mol Plant 2: 1154–1180. 19969518 10.1093/mp/ssp088

[48] Lenka SK, Lohia B, Kumar A, Chinnusamy V, Bansal KC (2009) Genome-wide targeted prediction of ABA responsive genes in rice based on over-represented *cis*-motif in co-expressed genes. Plant Mol Biol 69: 261–71. 18998058 10.1007/s11103-008-9423-4

[49] Saika H, Okamoto M, Miyoshi K, Kushiro T, Shinoda S, et al (2007) Ethylene promotes submergence-induced expression of *OsABA8ox1*, a gene that encodes ABA 8′-hydroxylase in rice. Plant Cell Physiol 48: 287–298. 17205969 10.1093/pcp/pcm003

[50] Yi J, An G (2013) Utilization of T-DNA tagging lines in rice. J Plant Biol 56: 85–90.

[51] Sakuraba Y, Yokono M, Akimoto S, Tanaka R, Tanaka A (2010) Deregulated chlorophyll *b* synthesis reduces the energy transfer rate between photosynthetic pigments and induces photodamage in *Arabidopsis thaliana* . Plant Cell Physiol 51: 1055–1065. 20403808 10.1093/pcp/pcq050

[52] Perrine Z, Negi S, Richard T, Sayre RT (2012) Optimization of photosynthetic light energy utilization by microalgae. Algal Res 1: 134–142.

[53] Ma B, Chen SY, Zhang JS (2010) Ethylene signaling in rice. Chinese Sci Bull 55: 2204–2210.

[54] Fang J, Chai C, Qian Q, Li C, Tang J, et al (2008) Mutations of genes in synthesis of the carotenoid precursors of ABA lead to pre-harvest sprouting and photo-oxidation in rice. Plant J 54: 177–189. 18208525 10.1111/j.1365-313X.2008.03411.x PMC2327239

[55] Pickett FB, Wilson AK, Estelle M (1990) The *aux1* mutation of *Arabidopsis* confers both auxin and ethylene resistance. Plant Physiol 94: 1462–1466. 16667854 10.1104/pp.94.3.1462 PMC1077399

[56] Luschnig C, Gaxiola RA, Grisafi P, Fink GR (1998) EIR1, a root-specific protein involved in auxin transport, is required for gravitropism in *Arabidopsis thaliana* . Genes Dev 12: 2175–2187. 9679062 10.1101/gad.12.14.2175 PMC317016

[57] Alonso JM, Stepanova AN, Solano R, Wisman E, Ferrari S, et al (2003) Five components of the ethylene-response pathway identified in a screen for weak ethylene-insensitive mutants in *Arabidopsis* . Proc Natl Acad Sci U S A 100: 2992–2997. 12606727 10.1073/pnas.0438070100 PMC151454

[58] Ruzicka K, Ljung K, Vanneste S, Podhorska R, Beeckman T, et al (2007) Ethylene regulates root growth through effects on auxin biosynthesis and transport-dependent auxin distribution. Plant Cell 19: 2197–2212. 17630274 10.1105/tpc.107.052126 PMC1955700

[59] Stepanova AN, Hoyt JM, Hamilton AA, Alonso JM (2005) A link between ethylene and auxin uncovered by the characterization of two root-specific ethylene-insensitive mutants in *Arabidopsis* . Plant Cell 17: 2230–2242. 15980261 10.1105/tpc.105.033365 PMC1182485

[60] Stepanova AN, Robertson-Hoyt J, Yun J, Benavente LM, Xie D, et al (2008) *TAA1*-mediated auxin biosynthesis is essential for hormone crosstalk and plant development. Cell 133: 177–191. 18394997 10.1016/j.cell.2008.01.047

[61] Stepanova AN, Yun J, Likhacheva AV, Alonso JM (2007) Multilevel interactions between ethylene and auxin in *Arabidopsis* roots. Plant Cell 19: 2169–2185. 17630276 10.1105/tpc.107.052068 PMC1955696

[62] Swarup R, Perry P, Hagenbeek D, Van Der Straeten D, Beemster GTS, et al (2007) Ethylene upregulates auxin biosynthesis in *Arabidopsis* seedlings to enhance inhibition of root cell elongation. Plant Cell 19: 2186–2196. 17630275 10.1105/tpc.107.052100 PMC1955695

[63] Vanstraelen M, Benkov E (2012) Hormonal interactions in the regulation of plant development. Annu Rev Cell Dev Biol 28: 22.1–22.25. 10.1146/annurev-cellbio-101011-155741 22856461

[64] Pacheco-Villalobos D, Sankar M, Ljung K, Hardtke CS (2013) Disturbed local auxin homeostasis enhances cellular anisotropy and reveals alternative wiring of auxin-ethylene crosstalk in *Brachypodium distachyon* seminal roots. PLoS Genet 9: e1003564. 23840182 10.1371/journal.pgen.1003564 PMC3688705

[65] Grossmanna K, Hansenb H (2001) Ethylene-triggered abscisic acid: A principle in plant growth regulation? Physiol Plant 113: 9–14.

[66] Hoffmann-Benning S, Kende H (1992) On the role of abscisic acid and gibberellin in the regulation of growth in rice. Plant Physiol 99: 1156–1161. 16668983 10.1104/pp.99.3.1156 PMC1080597

[67] LeNoble ME, Spollen WG, Sharp RE (2004) Maintenance of shoot growth by endogenous ABA: genetic assessment of the involvement of ethylene suppression. J Exp Bot 55: 237–245. 14673028 10.1093/jxb/erh031

[68] Ma B, Chen H, Chen SY, Zhang JS (2014) Roles of ethylene in plant growth and responses to stresses. In: Tran L-SP, Pal S, editors. Phytohormones: A window to metabolism, signaling and biotechnological applications. Springer Science+Business Media New York. pp. 81–118.

[69] Yang J, Zhang J, Wang Z, Liu K, Wang P (2006) Post-anthesis development of inferior and superior spikelets in rice in relation to abscisic acid and ethylene. J Exp Bot 57: 149–160. 16330527 10.1093/jxb/erj018

[70] Sharp RE, LeNoble ME, Else MA, Thorne ET, Gherardi F (2000) Endogenous ABA maintains shoot growth in tomato independently of effects on plant water balance: evidence for an interaction with ethylene. J Exp Bot 51: 1575–1584. 11006308 10.1093/jexbot/51.350.1575

[71] Trapnell C, Roberts A, Goff L, Pertea G, Kim D, et al (2012) Differential gene and transcript expression analysis of RNA-seq experiments with TopHat and Cufflinks. Nat Protocols 7: 562–578. 22383036 10.1038/nprot.2012.016 PMC3334321

[72] Fu J, Chu J, Sun X, Wang J, Yan C (2012) Simple, rapid, and simultaneous assay of multiple carboxyl containing phytohormones in wounded tomatoes by UPLC-MS/MS using single SPE purification and isotope dilution. Anal Sci 28: 1081–1087. 23149609 10.2116/analsci.28.1081

