# Supplementary figures and images for: Ethylene-Induced Inhibition of Root Growth Requires Abscisic Acid Function in Rice (Oryza sativa L.) Seedlings
Source: PLoS Genet. 2014 Oct 16;10(10):e1004701. doi: 10.1371/journal.pgen.1004701 (PMC4199509; doi:10.1371/journal.pgen.1004701)

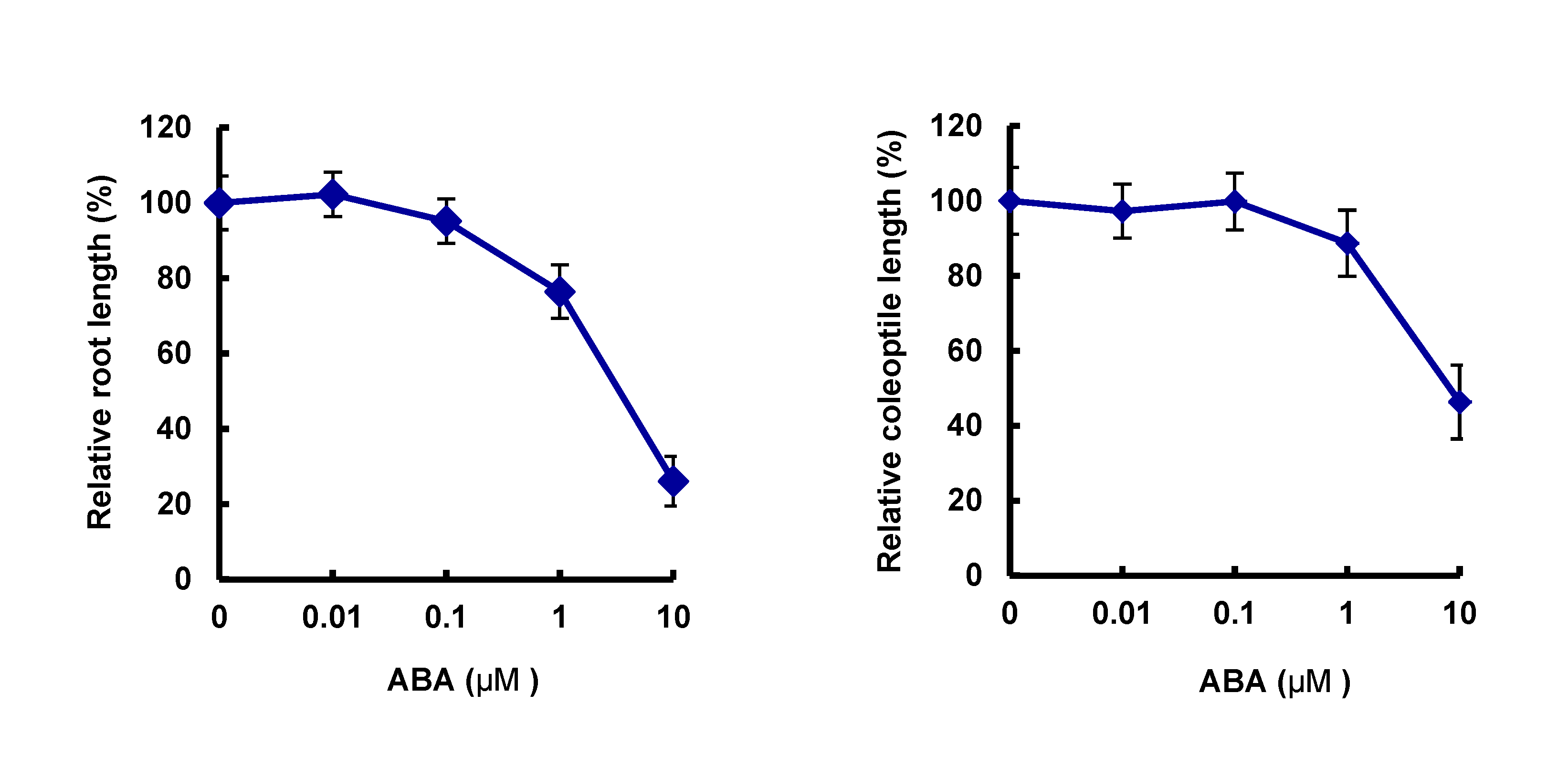

Supplement: Figure S1 — ABA dose-response curves for root and coleoptile growth in WT. Rice seedlings were grown in the dark for 3 d in the presence of various concentrations of ABA. Each point is average of 35 to 40 seedlings and bars indicate SD. (TIF) [file pgen.1004701.s001.tif]

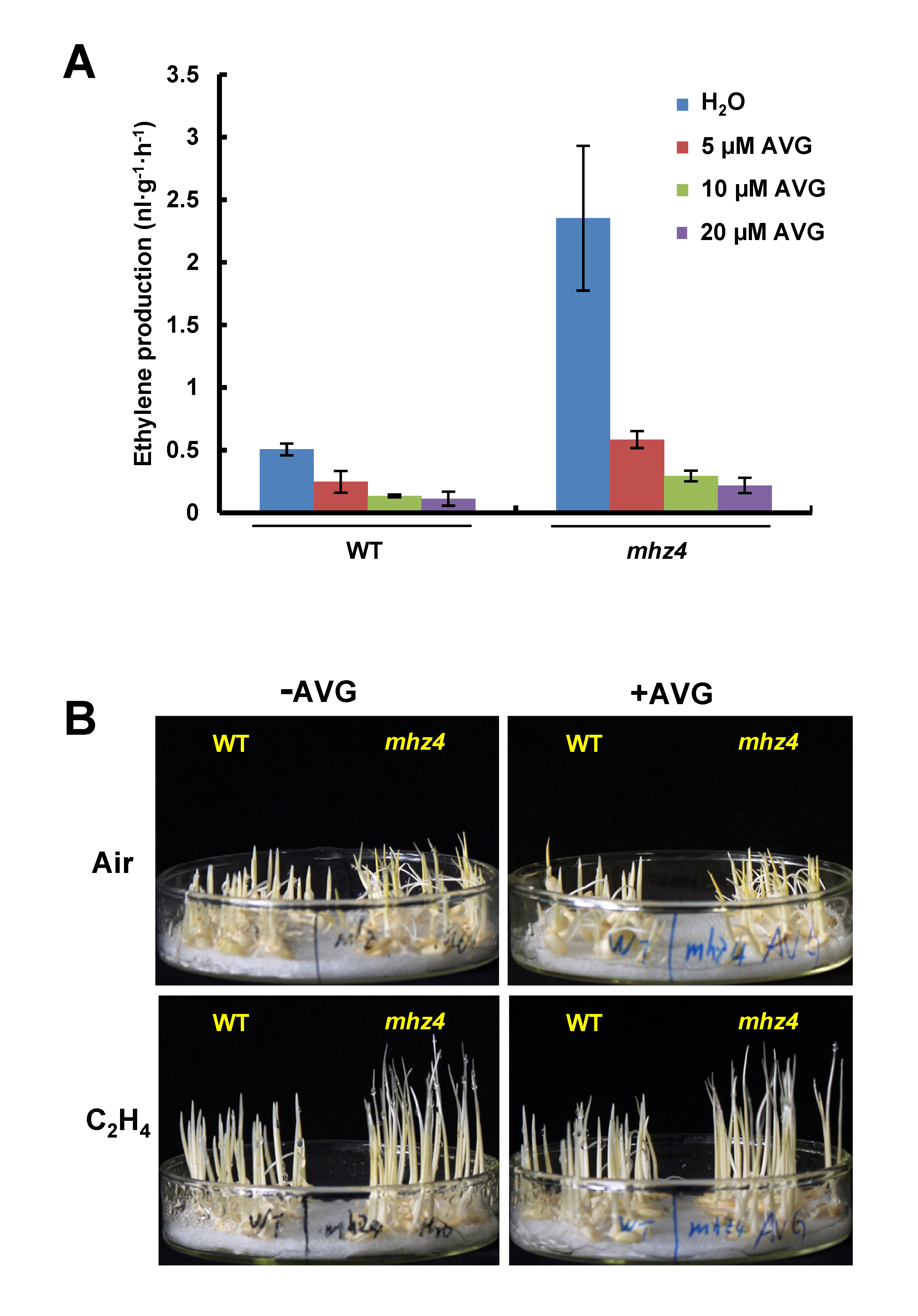

Supplement: Figure S2 — Effects of AVG treatment on ethylene production and coleoptile ethylene response of mhz4 and WT seedlings. (A) Ethylene production in the presence of various concentrations of AVG. Data are the mean ± SD of three replicates. (B) Coleoptile phenotypes of the seedlings grown in the dark for 2.5 days in the presence or absence of 10 ppm ethylene, supplemented with or without 5 µM AVG. (TIF) [file pgen.1004701.s002.tif]

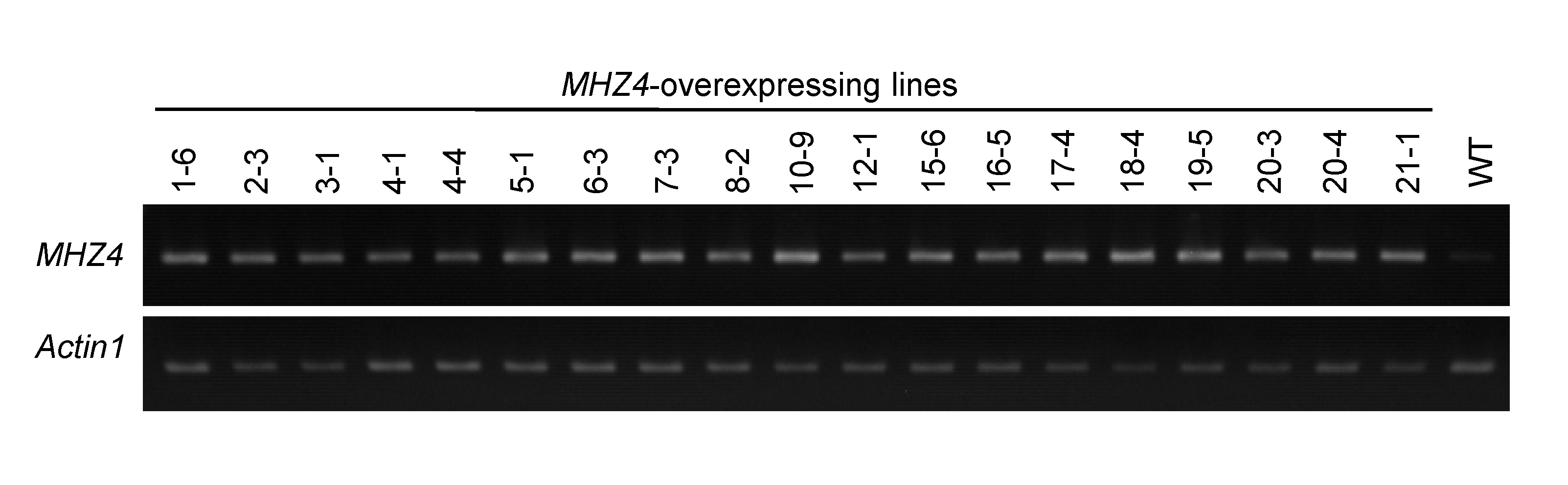

Supplement: Figure S3 — MHZ4 gene expression levels in overexpressing lines detected using semiquantitative RT-PCR. Actin1 was used as a control. (TIF) [file pgen.1004701.s003.tif]

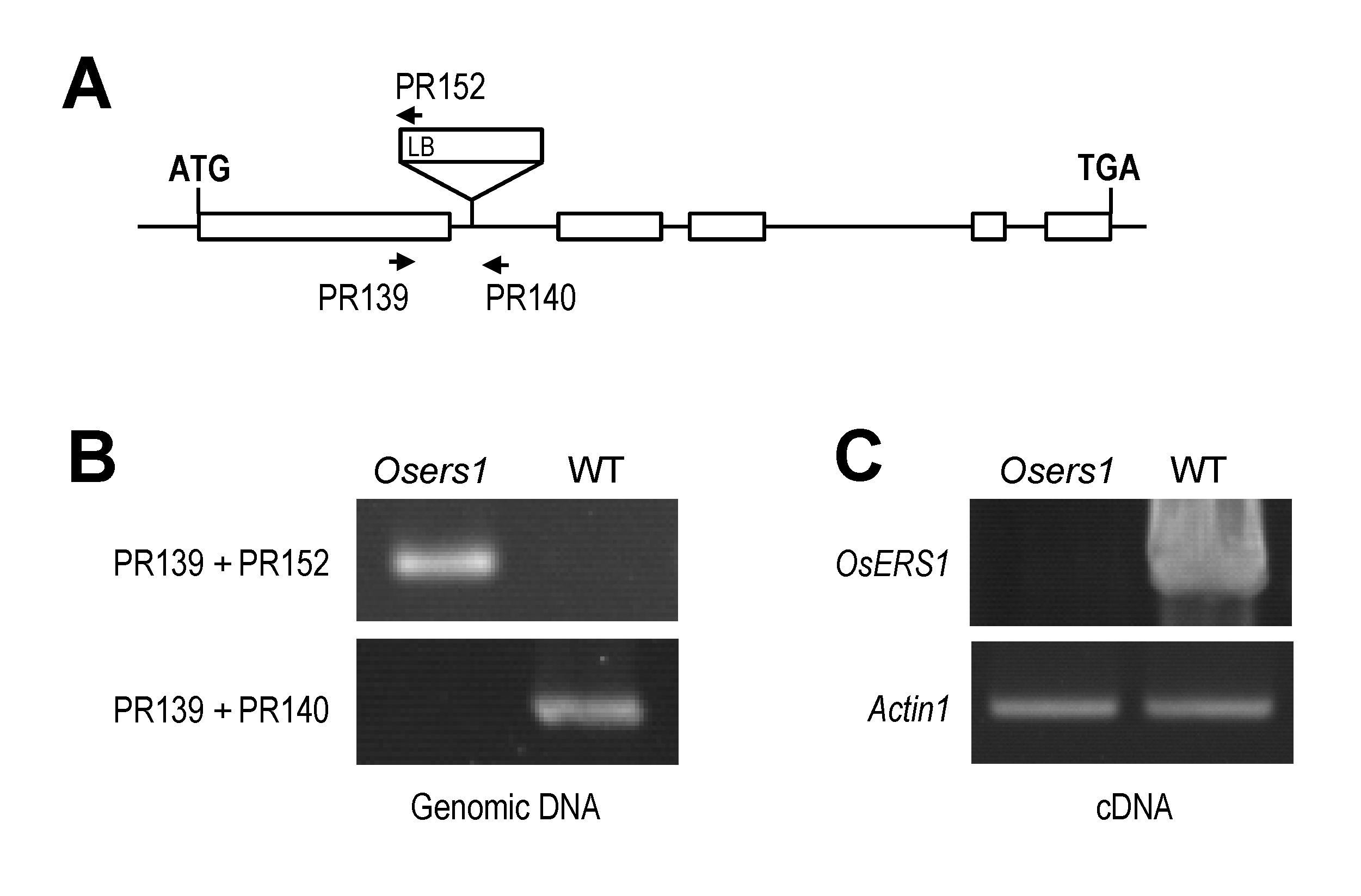

Supplement: Figure S4 — Identification of the Osers1 mutant. (A) Schematic representation of OsERS1 (LOC_Os03g49500) gene structure. The T-DNA insertion site and the primer positions are indicated. (B) PCR genotyping for the Osers1 mutant and WT (Dongjin). (C) OsERS1 expression in Osers1 and WT detected by RT-PCR analysis with amplification of the full-length cDNA. Actin1 was used as an internal control. (TIF) [file pgen.1004701.s004.tif]

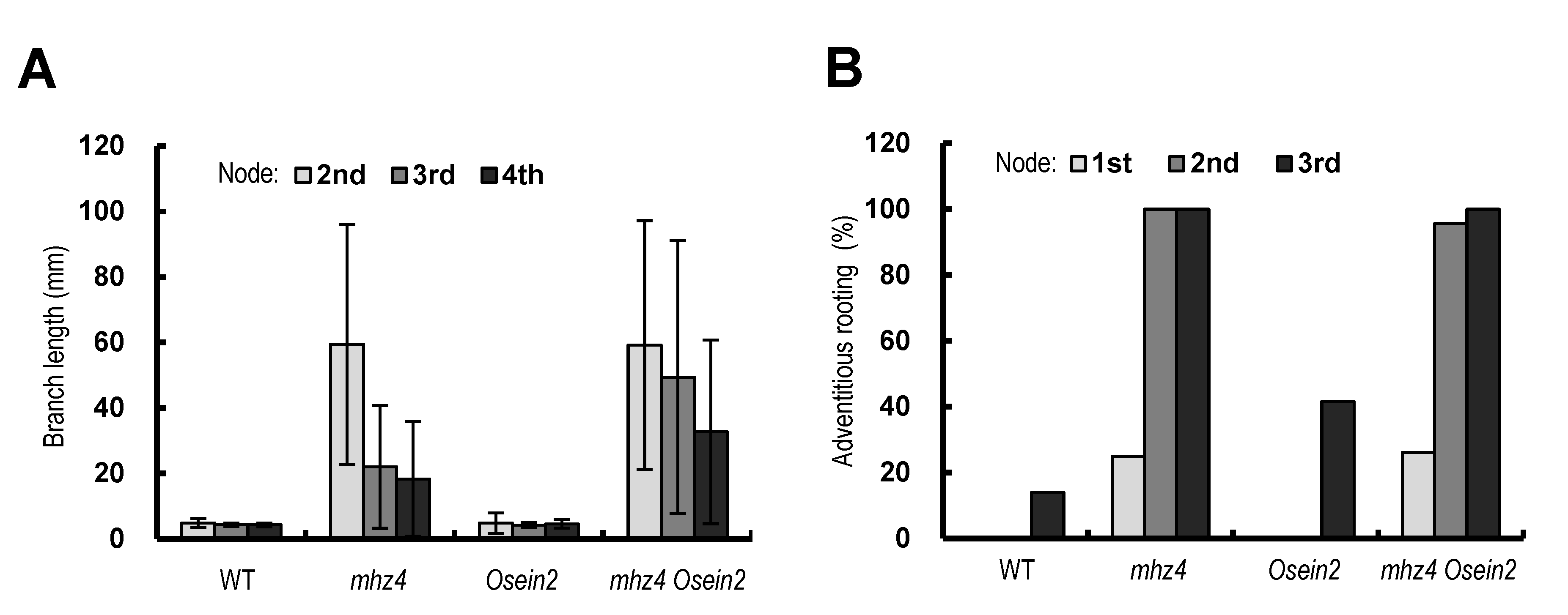

Supplement: Figure S5 — Quantification of branching and adventitious rooting in WT, mhz4, Osein2 and mhz4 Osein2 double mutant plants in a different year. (A) Branch length at each node in main tillers from 20 plants. (B) Percentage of adventitious root formation at each node in main tillers in 20 plants in (A). (TIF) [file pgen.1004701.s005.tif]

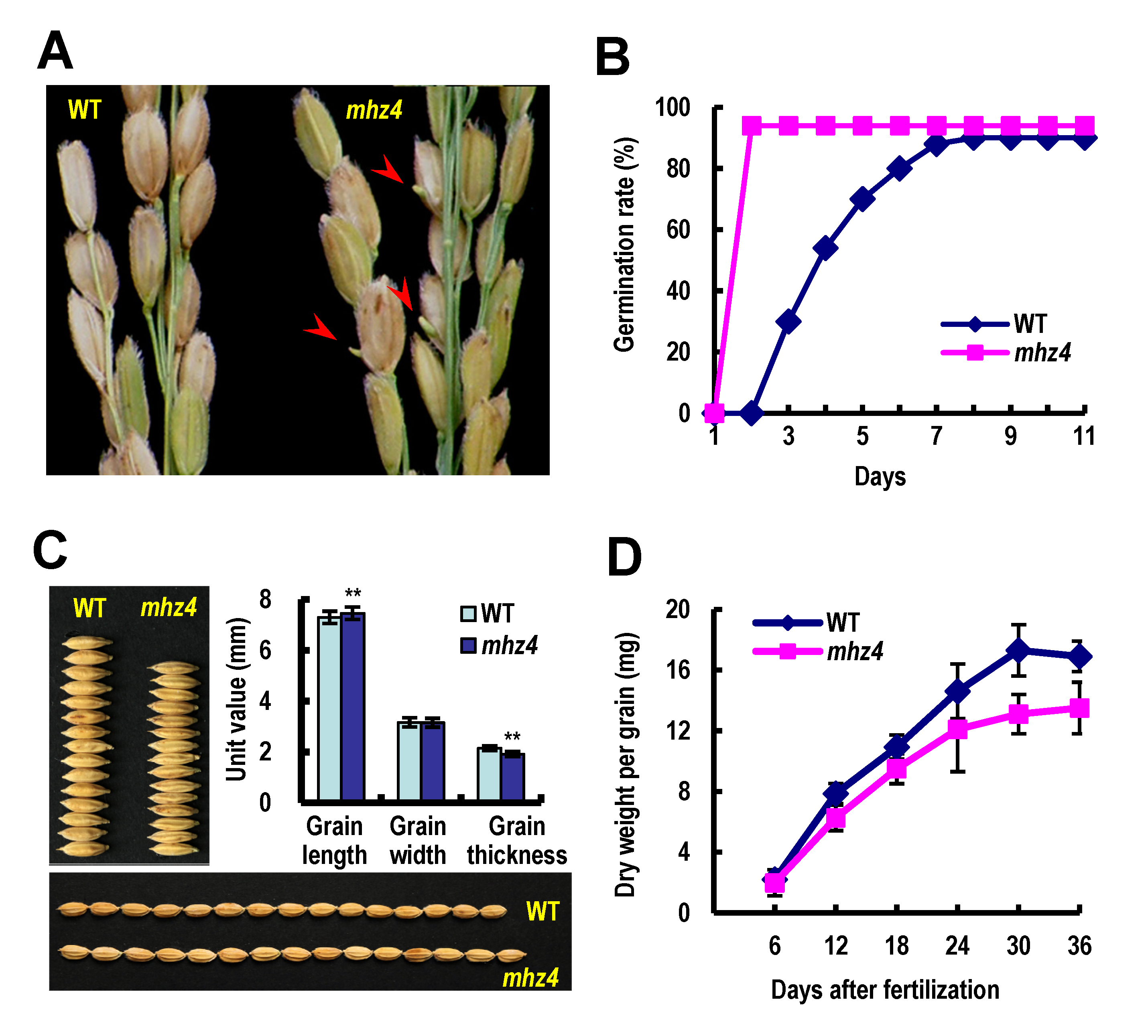

Supplement: Figure S6 — Preharvest sprouting and grain-related traits of mhz4 mutant. (A) Preharvest sprouting of mhz4. Arrow heads indicate germinated seeds on a panicle. (B) Germination rates of freshly harvested seeds of WT and mhz4. Each point is the percentage of germinated seeds among 100 seeds. (C) Comparison of grain thickness (top left panel), grain length (bottom panel) and their quantification results (top right panel) in WT and mhz4. Each column is an average of 100 grains and bars indicate SD. ‘**’ indicate significant difference compared to WT (P<0.01). (D) Time-course of grain-filling after fertilization. Each point is an average of 50–100 grains and bars indicate SD. (TIF) [file pgen.1004701.s006.tif]
