# Supplementary material for: Ethylene-Induced Inhibition of Root Growth Requires Abscisic Acid Function in Rice (Oryza sativa L.) Seedlings
Source: PLoS Genet. 2014 Oct 16;10(10):e1004701. doi: 10.1371/journal.pgen.1004701 (PMC4199509; doi:10.1371/journal.pgen.1004701)
Supplement: Table S1 — Primers used gene expression analysis and plasmid construction. (DOCX) [file pgen.1004701.s007.docx]

**Table S1.** Primers used gene expression analysis and plasmid construction.

| Genes or constructs |  | Locus ID | Forward primer (5’-3’) | Reverse primer (5’-3’) |
| --- | --- | --- | --- | --- |
| **Real-time PCR** |  |  |  |  |
| *MHZ4* |  | LOC_Os01g03750 | ctctgttccggcctcgcgca | tcaatgtgagcgaccaattgaac |
| *SHR5* |  | LOC_Os08g10310 | aataccagctatgttaccagcc | caccattacaaattacaaggagc |
| *Disease resistance response gene* |  | LOC_Os10g18820 | ctggccaaagcaatgctaac | tggcagacgagtagcatttg |
| *Peptidylprolyl isomerase gene* |  | LOC_Os01g38359 | actagtggtcttgctacacc | tacgttaacccaataaagggat |
| *OsERF063* |  | LOC_Os09g11480 | acgtgatggacagcctcctc | gggaagtctgaaatggacatg |
| *OsERF073* |  | LOC_Os09g11460 | aatgataatcaaggcaccac | acccgaataagtgttgataac |
| *Photosystem II 10 kDa polypeptide* |  | LOC_Os07g05360 | aacatggctgcctctgtcatggc | gcaggcccatagggctgggat |
| *OsRRA5* |  | LOC_Os07g26720 | tgggctcggaacctaatgtg | acgacattatcaccaccggg |
| *OsRAP2.8* |  | LOC_Os11g05740 | gagctcctatgctgccatgt | ggcactatggggatggaagg |
| *OsIAA20* |  | LOC_Os06g07040 | gctcatacgctgagctgctcgac | cacgtctccgacctgcatccag |
| *OsERF002* |  | LOC_Os06g08340 | gcagtacgtggaccagatgatc | ctcgatcagagttcttcctcac |
| *OsACS2* |  | LOC_Os04g48850 | tttggcgccttgacggcctc | aaagggagcgcaccatggcc |
| *OsACS6* |  | LOC_Os06g03990 | ccgggcgacacgttcagctt | acagcgcgaacgggttccag |
| *OsACO3* |  | LOC_Os09g27750 | tgtccctgtcccacccaggg | cgtaagctcagcgatgaaatc |
| *OsACO5* |  | LOC_Os05g05680 | tgcaacagcacgccacacca | tggatcgacgtccagcccgt |
| *Actin2* |  | LOC_Os10g36650 | ttatggttgggatgggaca | agcacggcttgaatagcg |
| **Semiquantitative-PCR** |  |  |  |  |
| *OsMFT2* |  | LOC_Os01g02120 | gtggtggatttgttcgttcc | cgcgaatgctcttgtgttga |
| *OsEIN2* |  | LOC_Os07g06130 | cagaactttggggcaagtat | tattgttgtcccttgctcgagagc |
| *Actin1* |  | LOC_Os03g50885 | tccatcttggcatctctcag | gtaccctcatcaggcatctg |
| **Constructs** |  |  |  |  |
| *MHZ4-GFP* |  |  | tatctcgagatggcggctctcctcctcct | gcaccatggaatgtgagcgaccaattgaac |
| *MHZ4pro*::*GUS* |  |  | gacctgcagggtgtgaaagcaatgcaagaa | taggatccagggcaggaagcgagcagga |
| *MHZ4*-complementation |  |  | gacctgcagggtgtgaaagcaatgcaagaa | agactcgagtcctgctcaaacacttccac |
| *35S::MHZ4* |  |  | ttaagatctgcaatggcggctctcctcct | cgtctcgagtcaatgtgagcgaccaattg |
| *NPT II* |  |  | atggggattgaacaagatggatt | tcagaagaactcgtcaagaagg |
