# Supplementary material for: Ethylene-Induced Inhibition of Root Growth Requires Abscisic Acid Function in Rice (Oryza sativa L.) Seedlings
Source: PLoS Genet. 2014 Oct 16;10(10):e1004701. doi: 10.1371/journal.pgen.1004701 (PMC4199509; doi:10.1371/journal.pgen.1004701)
Supplement: Table S2 — Yield-related traits in WT, mhz4 mutant and MHZ4-overexpressing lines. Each value is average of 20 to 24 individuals. * and ** indicate significant difference compared to WT at P<0.05 and P<0.01, respectively. Data from two years are presented. (DOCX) [file pgen.1004701.s008.docx]

**Table S2.** Yield-related traits in WT, *mhz4* mutant and *MHZ4*-overexpressing lines. Each value is average of 20 to 24 individuals. * and ** indicate significant difference compared to WT at P<0.05 and P<0.01, respectively. Data from two years are presented.

|  | Year | Heading  date ± SD | Plant height (cm)± SD | Effective tiller number  ± SD | Panicle length (cm) ±  SD | Number of grains per panicle ± SD | Seed setting rate (%) ± SD | 1,000-  grain weight (g)±SD | Grain weight (g) per plant ± SD |
| --- | --- | --- | --- | --- | --- | --- | --- | --- | --- |
| WT | Year1 | 107.9  ± 1.3 | 93.6  ± 2.5 | 7.9  ± 1.5 | 19.3  ± 0.8 | 92.0  ± 10.6 | 81.6  ± 11.3 | 22.9  ± 1.3 | 13.5  ± 3.1 |
|  | Year2 |  | 100.4  ± 2.2 | 11.6  ± 2.1 | 19.3  ± 0.8 | 85.0  ± 6.8 | 89.0  ± 8.0 | 22.8  ± 1.1 | 19.8  ± 3.4 |
| *mhz4* | Year1 | 112.3 **  ± 1.4 | 95.7  ± 4.3 | 8.6  ± 1.9 | 18.3**  ± 0.8 | 95.4  ± 10.0 | 37.0**  ± 12.4 | 16.1**  ± 0.6 | 4.8**  ± 2.0 |
|  | Year2 |  | 105.9**  ± 1.9 | 11.5  ± 3.8 | 18.2**  ± 0.9 | 89.1  ± 11.6 | 65.7**  ± 9.2 | 15.1**  ± 0.9 | 10.0**  ± 3.4 |
| OX7-3 | Year1 | 110.6 **  ± 1.3 | 83.2 **  ± 4.2 | 8.5  ± 1.8 | 19.3  ± 0.8 | 91.5  ± 12.9 | 70.8  ± 10.5 | 20.0**  ± 1.3 | 11.4  ± 4.8 |
|  | Year2 |  | 84.3 **  ± 2.6 | 11.8  ± 2.4 | 18.9  ± 0.8 | 71.4**  ± 8.2 | 73.0**  ± 4.8 | 17.3**  ± 1.0 | 10.7**  ± 2.6 |
| OX8-2 | Year1 | 109.8**  ± 1.2 | 85.4**  ± 4.1 | 9.5*  ± 2.3 | 18.8  ± 0.8 | 103.4**  ± 8.7 | 72.7  ± 10.1 | 18.7**  ± 1.2 | 13.5  ± 4.5 |
|  | Year2 |  | 94.3**  ± 4.5 | 13.1  ± 2.8 | 18.9  ± 0.9 | 83.8  ± 12.5 | 81.8**  ± 2.7 | 16.0**  ± 1.5 | 18.1  ± 7.1 |
| OX10-9 | Year1 | 110.2**  ± 1.2 | 89.0**  ± 2.1 | 9.9**  ± 1.4 | 19.1  ± 0.7 | 89.8  ± 5.7 | 82.9  ± 3.6 | 20.0**  ± 1.3 | 14.7  ± 2.5 |
|  | Year2 |  | 95.7**  ± 3.1 | 12.1  ± 2.3 | 19.4  ± 1.3 | 77.8*  ± 13.2 | 84.0*  ± 4.0 | 19.4**  ± 0.7 | 15.5**  ± 4.7 |
| OX20-3 | Year1 | 110.4**  ± 1.2 | 86.0**  ± 2.0 | 8.3  ± 1.9 | 19.0  ± 0.7 | 104.0**  ± 26.4 | 84.7  ± 3.3 | 21.5*  ± 0.8 | 18.6**  ± 4.5 |
|  | Year2 |  | 97.7**  ± 2.3 | 11.9  ± 2.1 | 18.5**  ± 1.0 | 86.6  ± 10.5 | 86.4  ± 2.9 | 19.8**  ± 1.2 | 17.9  ± 5.1 |
| *Osein2* | Year1 |  | 78.2**  ± 3.7 |  |  |  |  |  |  |
|  | Year2 |  | 93.8**  ± 1.7 |  |  |  |  |  |  |
| *mhz4 Osein2* | Year1 |  | 73.0**  ± 3.6 |  |  |  |  |  |  |
|  | Year2 |  | 92.0**  ± 1.7 |  |  |  |  |  |  |
